# Pesticide concentrations in agricultural storm drainage inlets of a small Swiss catchment

**DOI:** 10.1007/s11356-022-18933-5

**Published:** 2022-02-05

**Authors:** Urs T. Schönenberger, Birgit Beck, Anne Dax, Bernadette Vogler, Christian Stamm

**Affiliations:** Eawag, Swiss Federal Institute of Aquatic Science and Technology, 8600 Dübendorf, Switzerland

**Keywords:** Storm drainage inlets, Hydraulic shortcuts, Field study, Agricultural runoff, Surface runoff, Spray drift, Pesticide concentrations, Pesticide transport pathways

## Abstract

Agricultural pesticides transported to surface waters pose a major risk for aquatic ecosystems. Modelling studies indicate that the inlets of agricultural storm drainage systems can considerably increase the connectivity of surface runoff and pesticides to surface waters. These model results have however not yet been validated with field measurements. In this study, we measured discharge and concentrations of 51 pesticides in four out of 158 storm drainage inlets of a small Swiss agricultural catchment (2.8 km^2^) and in the receiving stream. For this, we performed an event-triggered sampling during 19 rain events and collected plot-specific pesticide application data. Our results show that agricultural storm drainage inlets strongly influence surface runoff and pesticide transport in the study catchment. The concentrations of single pesticides in inlets amounted up to 62 µg/L. During some rain events, transport through single inlets caused more than 10% of the stream load of certain pesticides. An extrapolation to the entire catchment suggests that during selected events on average 30 to 70% of the load in the stream was transported through inlets. Pesticide applications on fields with surface runoff or spray drift potential to inlets led to increased concentrations in the corresponding inlets. Overall, this study corroborates the relevance of such inlets for pesticide transport by establishing a connectivity between fields and surface waters, and by their potential to deliver substantial pesticide loads to surface waters.

**Supplementary Information:**

The online version contains supplementary material available at 10.1007/s11356-022-18933-5.

## Introduction

Pesticides used in agriculture impair water quality, leading to biodiversity losses in aquatic ecosystems and threaten drinking water resources (Stehle and Schulz, 2015; Sánchez-Bayo and Wyckhuys, 2019; Kiefer et al., 2020). To protect surface waters from those negative impacts with appropriate measures, it is essential to understand how pesticides are transported from the field to surface waters. Current research usually distinguishes between two types of pesticide transport pathways: point sources and diffuse sources. Farmyard runoff (De Wilde et al., 2007; Reichenberger et al., 2007), wastewater treatment plants (Eggen et al., 2014; Munz et al., 2017), combined sewer overflows (Neumann et al., 2002; Mutzner et al., 2020) or accidental spills (Reichenberger et al., 2007) are considered the most important point sources. For diffuse sources, surface runoff (Larsbo et al., 2016; Lefrancq et al., 2017), spray drift (Vischetti et al., 2008; Lefrancq et al., 2013) and macropore flow to tile drainages (Sandin et al., 2018) are considered of major importance.

Pesticide transport from diffuse sources has been shown to be strongly influenced by artificial structures affecting the connectivity between fields and the stream network (Frey et al., 2009). For example, in several studies, roads and ditches were shown to concentrate surface runoff and increase pesticide losses (Rübel, 1999; Heathwaite et al., 2005; Payraudeau et al., 2009; Fiener et al., 2011; Hösl et al., 2012). Additionally, in a French vineyard, spray drift on roads and subsequent wash off was found to be a major pesticide transport pathway (Lefrancq et al., 2014). In contrast to other countries, roads and adjacent fields in Switzerland are less often drained to ditches, but to inlet and maintenance shafts of storm and tile drainage systems (Alder et al., 2015). In a model-based study on the national level, we found that around half of surface runoff from fields and the related pesticide load is expected to be transported to surface waters through such shafts (Schönenberger and Stamm, 2021; Schönenberger et al. 2022). Similarly, another model-based study suggests that also the wash-off of spray drift deposited on roads through such shafts to surface waters may be a major pesticide transport pathway (Schönenberger et al., 2022).  However, there is a lack of empirical data to validate these findings. So far, field data on transport of agricultural pollutants through inlet or maintenance shafts were only reported in two studies. Firstly, Remund et al. (2021) performed a long-term study on soil erosion in five Swiss study catchments. They found that 88% of the sediment and phosphorus losses from arable land to surface waters occurred through inlet or maintenance shafts. Secondly, Doppler et al. (2012) measured pesticide concentrations in the stream and the underground pipe system of a small Swiss agricultural catchment. They found that inlet shafts, maintenance shafts and the connected pipe system were creating shortcuts between remote areas of the catchment and the stream, enabling fast transport of surface runoff and pesticides. Inlet and maintenance shafts were therefore called hydraulic shortcuts.

**Fig. 1:**
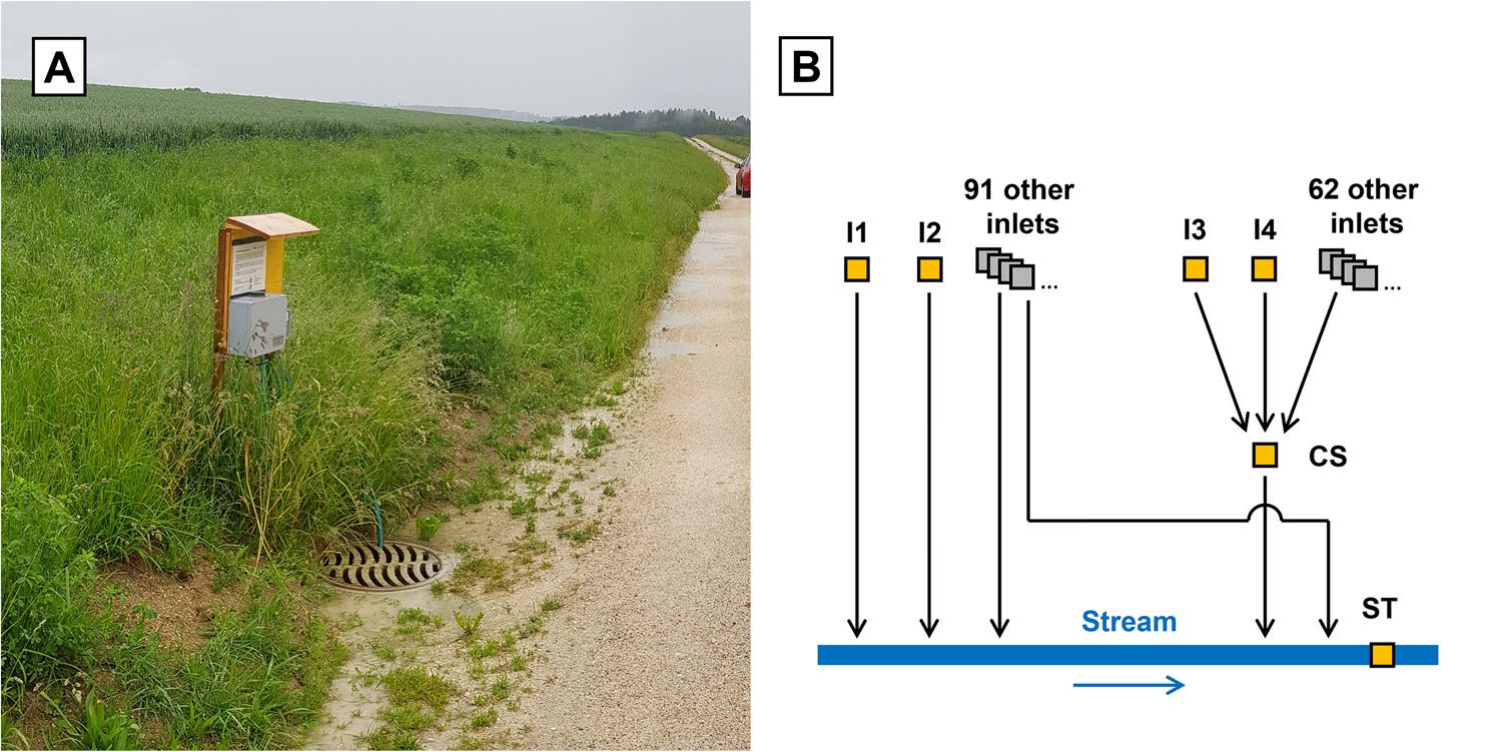
**A** Example picture of a storm drainage inlet in the study catchment taken during the study period. The depicted inlet (I1) is one of the four inlets sampled and is situated between a farm track and a wheat field. A larger picture of the situation around the inlet is shown in Figure A1. **B** Schematic representation of the storm drainage network in the catchment (black lines: pipes, grey squares: inlets) and of the sampling locations (yellow squares). I1–I4: inlets; CS: collector shaft; ST: stream

Although the above-mentioned studies indicate that hydraulic shortcuts can be a relevant transport pathway, direct measurements of surface runoff and pesticides transported through hydraulic shortcuts in agricultural areas currently do not exist. To close this gap, we measured runoff and pesticide transport through inlet shafts (or simply inlets in the following, see Fig. 1A) of an agricultural storm drainage system for the first time. The measurements were performed in a catchment in which we expected rather high pesticide transport through hydraulic shortcuts (i.e. an intensively used agricultural catchment with a high shortcut density). We focussed our study on inlets, since this type of hydraulic shortcut was identified as the most important shortcut type in a previous study (Schönenberger and Stamm, 2021).

Therefore, we aimed on answering the following research questions:

- 1. How often is surface runoff transported through storm drainage inlets and which ratio of the discharge in the stream is caused by this process?
- 2. Which pesticide concentrations and loads are transported during selected rain events?
- 3. How are transport pathways, pesticide applications and substance properties affecting pesticide concentrations in inlets?

To answer these questions, we focused on a study catchment with a high number of shortcuts and little direct surface connectivity to the stream. However, the conditions in the study catchment (soils, topography, climate, storm drainage system) are quite typical for the Swiss Plateau such that key findings can be generalised to a larger area.

## Material and methods

### Study catchment

**Fig. 2:**
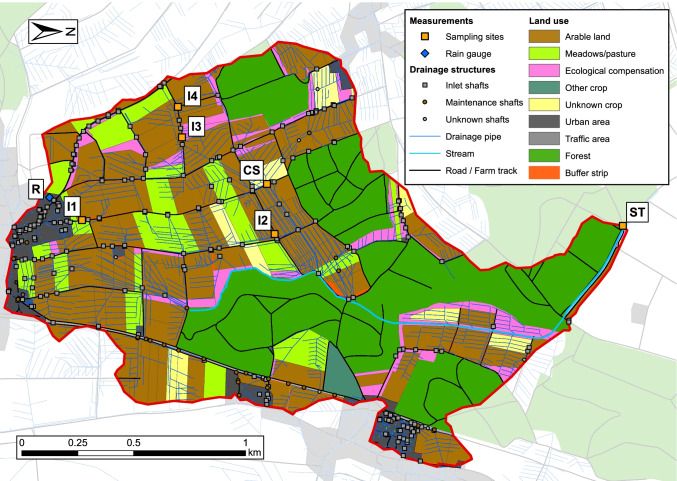
Map of the study catchment. Yellow squares show the sampling sites (I1–I4: inlets; CS: collector shaft; ST: stream) and the blue diamond shows the rain gauge (R)

The study catchment (Fig. 2) is located in a rural area in the Swiss midlands (canton of Bern, outlet: 47°07′12.570″N 7°30′48.926″E). It has a size of 2.8 km^2^ and is covered by arable land (38%), forests (32%), agricultural areas with very little or no pesticide use (18%) (e.g. meadows, pasture, ecological compensation areas) and other/undefined agricultural areas (4%). Settlements, farmyards, roads and farm tracks mainly cover the remaining area (8%). On arable land, the predominant crop types during the study year were grains, potatoes, and sugar beets. The average annual rainfall equals 1075 ± 163 mm/year (MeteoSwiss, 2018) and the average slope is 5.0%. The agricultural area is heavily drained by artificial structures by tile drains in the soils and by storm drains along the road network. In total, 158 storm drainage inlets (see Fig. 1A) were identified along or on agricultural areas. Most of them are located along farm tracks (111), or concrete roads (33). The remaining fourteen are located directly on fields. All of these inlets are drained to the stream at the catchment outlet. In addition, 84% of the agricultural area is tile drained.

Most of the 26 farmers in the catchment were participating in a programme aiming to reduce pesticide pollution in the receiving stream. They had the freedom to decide on pesticide applications themselves, but received subsidies for reduction measures (e.g. creating buffer strips or reducing herbicide use). We received plot-specific crop and pesticide application data for 96% of the agricultural areas in the catchment for the period January to October of the study year 2019. The pesticide application data was recorded by the farmers using a crop management system and included the day of application, product, amount applied, crop, plot size and a georeferenced polygon of the plot. Source of background map: Swisstopo (2020)

### Field work

#### Sampling site selection

We selected six sampling sites in the catchment (see Fig. 1B and Fig. 2). Four were located at storm drainage inlets (I1–I4), and one each at a collector shaft (CS) and the stream (ST) at the catchment outlet.

I1–I4 were selected as follows from the 158 inlets in the catchment. To be a suitable sampling location, an inlet had to fulfil two criteria. First, the dimensions of the inlet had to allow the installation of measuring equipment. Second, we aimed on sampling only surface runoff entering the inlet through the lid, but no other inflows. To ensure that no tile drainage flow enters the inlet, we therefore also excluded all inlets with inflow pipes. From the ten inlets fulfilling these criteria, we selected the four that represented the different terrain and cropping conditions best (see Figure A1 to Figure A4). They are all located at the border of a field and a gravel farm track. While I1, I2 and I4 are lying directly next to the farm track, I3 is separated from the farm track by a grass strip of approximately 0.5-m width (Figure A3). During dry periods, there is no discharge transported through the four inlets, and in I1, I2 and I4 the water stagnates at the height of the outlet pipe (Figure A5). In contrast, during dry periods, the water level in I3 falls to a lower level due to seepage through the shaft bottom.

Because of the second selection criterion, the selected inlets only cover a small fraction of the total surface runoff transported through storm drainage inlets in the catchment. By measuring in shafts collecting storm drainage water from several inlets, we could have increased the fraction of surface runoff sampled. However, in most shafts, it was not possible to distinguish if an inflow pipe is only connected to storm drainage inlets, or also to the tile drainage system. The restriction of our measurements to inlets without inflow pipes was therefore necessary to ensure that our signal only consists of surface runoff.

#### Installations

##### *Inlets (I1–I4)*

In each inlet, we measured discharge by installing a weir with a calibrated rating curve in front of the outlet pipe. The water level was measured using a capacitive pressure sensor (DWL compact, UIT, Germany) coupled to a data logger equipped with a GPRS module (LogTrans-field, UIT, Germany). For water sampling, we installed an event-based, water-level proportional sampler (details in Section A1.1.2). The GPRS module was used for triggering other samplers (details in the “Sampling strategy” section), and data transfer, and to inform scientists.

##### *Collector shaft (CS)*

This shaft collects water from 64 inlets (including I3 and I4), and from a large part of the tile drainage system in the catchment (Figure A6 and Figure A7). At this site, the water level was measured using the same sensors as in the inlets. Water samples were taken using an automatic sampler (TP5C portable sampler, MAXX GmbH, Germany) coupled to a GPRS module.

##### *Stream (ST)*

At the catchment outlet (Figure A8), discharge was measured by the cantonal authorities using an ultrasonic sensor (POA-V2XXK, NIVUS AG, Germany). Water samples were taken with the same sampler type as in the collector shaft.

##### *Rain gauge (R)*

Rainfall data (resolution: 1 min, accuracy: 0.1 mm) was provided by the cantonal authorities from a rain gauge at the southern catchment edge.

#### Sampling strategy

In central Europe, most pesticides are applied in spring and summer (Szöcs et al., 2017; Halbach et al., 2021) and rainfall intensities are higher during this time of the year (Umbricht et al., 2013). Consequently, the highest pesticide concentrations in surface waters are usually measured during this period (Doppler et al., 2017). We therefore selected our study period (1 April to 20 August 2019) such that it covers most of this high-risk period. From the substances analysed in this study, 96% of the total active ingredient mass applied in 2019 was applied within this period (see Figure A11). Since water only flows through the inlets during rain events, we performed an event-based sampling.

In the inlets, the water-level proportional samplers started sampling at a defined water level threshold above the bottom of the weir (2 cm for inlets with little runoff, 3 cm for inlets with larger runoff), corresponding to a discharge of approximately 1.7 and 5 L/min. This resulted in one composite sample per event for each inlet exceeding the water level threshold. Rain events that were too small to exceed the water level threshold in an inlet were not sampled. When the water level threshold was exceeded in at least two inlets, the automatic samplers at the collector shaft and the stream were triggered via the GPRS module to start sampling (see Figure A10). In the collector shaft, time proportional samples (50 mL) were taken every 2 to 3 min and pooled together into one composite sample per 20 to 30 min, depending on the event (details in Table A6). Depending on the event duration, the total sampling duration was 4 to 8 h. In the stream, time proportional sampling was performed with the same frequency during the discharge peak. Before and after the peak, samples were pooled over a period of up to 2 h. Depending on the event duration, the total sampling duration was 10 to 12 h. All samples were kept in glass bottles and protected from sunlight. At sites CS and ST, the samples were cooled by the automatic samplers (4 °C), and at sites I1–I4 by the stagnating water around the bottle (average temperature: 13.5 °C). They were collected on average 1.3 days after sampling and frozen at −20 °C until analysis. If no composite samples were taken in an inlet during an event (due to lack of sufficient discharge, or due to malfunctioning of the sampler), we took a grab sample from the stagnant water during sample collection. Cantonal authorities were also taking samples in the stream (15-min sampling interval, 8 h composite samples) serving as a backup in case of malfunctioning of the automatic sampler.

#### Field mapping

During a snowmelt event on 12 March 2018, we mapped the surface runoff pathways in a part of the catchment (Figure A12). We chose a snowmelt event for this mapping campaign, since it was easier to predict snowmelt events than intense rainfall events generating surface runoff. Since runoff pathways strongly depend on the amount of runoff formed, this mapping campaign only provides a rough estimation of the potential runoff pathways during rain events.

### Chemical analysis

Overall, we collected 423 samples and selected 193 of them as the most relevant ones (see below) for further analysis. Most importantly, we analysed all inlet samples. In a second step, we analysed collector shaft and stream samples for six out of the top ten events with the highest sum concentrations in the sampled inlets, such that they cover the range of rain intensities observed (details in Table A6). For the selected samples, dissolved phase pesticide concentrations were determined using direct injection liquid chromatography coupled to high-resolution mass spectrometry (LC-HRMS). The particulate phase was not analysed. The target list (Table A3) included 51 substances that were either pesticides known to be applied in the catchment (45 substances) or their transformation products (6 substances). Samples were thawed and centrifuged for 5 min at 2000* g*. The supernatant was transferred and isotope-labelled internal standard (ISTD) was spiked (details in Table A3). Randomly selected samples were spiked with a standard solution in order to assess relative recovery of the compounds. Centrifugation, transfer, spiking of ISTD and standard solution were performed by a fully automated workflow. Laboratory blanks and blinds, and field blinds, were included in the measurement sequence to monitor instrument carry-over and contamination. Chromatographic separation was performed on a reversed-phase C18 column (Atlantis T3, 3-µm particle size, 3.0 × 150 mm inner diameter, Waters), applying a water–methanol gradient (both containing 0.1% formic acid). The measurements were performed on a hybrid quadrupole-orbitrap mass spectrometer (Lumos Fusion, Thermo Scientific) equipped with an electrospray ionisation source. Quantification of the target compounds was performed using TraceFinder 5.1 (Thermo Scientific). For 95% of the compounds, relative recovery was in the range of 80–120%. For 80% of the compounds, the limit of quantification (LOQ) was 20 ng/L or lower. Further details on the chemical analysis (such as the gradient, the ionisation, processed sample volumes) are given in Section A1.2

### Data analysis

#### Surface runoff connectivity

To determine the topographical catchment of each sampling site with respective crops and pesticide applications, we used a surface runoff connectivity model (Schönenberger and Stamm, 2021). The model is based on a digital elevation model (Swisstopo, 2019) with 2 × 2 m resolution and a D-infinity flow algorithm (Tarboton, 1997). Despite the high spatial resolution, it cannot represent all microtopographical features such as subtle depressions or the effects of roads. These sub-grid effects are represented by average effects in the model parameterisation. We adjusted the model parameters (e.g. road carving depth, or sink filling depth) such that the output fitted the observed flow paths in the field well (details in Table A 1).

The model output indicates from which agricultural areas (called contributing areas in the following) surface runoff drains to a particular inlet or directly to the stream, and from which areas surface runoff infiltrates in a sink. We intersected the contributing areas with the plot-specific crop and pesticide application data. This provided us with an estimate of crops planted and pesticides applied in the contributing area of each inlet, sink and the stream.

In addition, we performed a Monte Carlo simulation of the surface runoff connectivity model with 100 model runs. The parametrization was identical as in Schönenberger and Stamm (2021). This allowed us to assess the uncertainty introduced by the model parameter selection, and to compare the connectivity in the study catchment to the national assessment of the mentioned study.

#### Definition of events

We classified two types of events — rainfall and sampling events. Measured rainfall was classified into a rainfall event if the total rainfall exceeded 1 mm within 8 h. Subsequent rainfall was assigned to the same event if there was no dry period of at least 8 h in between. After dry periods of more than 8 h, a new rainfall event was defined. Sampling events were defined as rainfall events during which water samples were taken.

#### Transport processes

For each measured pesticide in a sample, we determined potential transport processes causing the measured concentration. Based on the spatio-temporal relation between samples and applications, we assigned each concentration measurement to one of the following categories: (A) No reported application, (B) other, (C) spray drift/other, (D) surface runoff/(tile drainage)/spray drift/other. In the following, we explain these categories and how they were assigned.

- *A) No reported application*. If the pesticide was not applied in the catchment during the study year, or only after the sample was taken, the measured concentration was assigned to this category. Concentrations in this category may be due to wash-off of residuals from previous year’s applications, originate from unreported applications or relate to applications outside the study catchment (e.g. atmospheric deposition).
- *B) Other.* This category was assigned if the pesticide was applied in the catchment before the sampled event, but on a field not allowing for transport via spray drift, surface runoff or tile drainages to the sampling site. Concentrations in this category may originate from droplet losses from leaky spraying equipment, farmyard runoff, accidental spills, atmospheric deposition or a process mentioned in the previous category.
- *C) Spray drift/other*. This category was assigned if the pesticide was applied before the event and spray drift to the sampling site was possible, but not transport via surface runoff or tile drainages. In the study catchment, only ground applications are performed and spray drift may reach the site in two ways: Firstly, spray drift can directly be deposited in the inlet, the collector shaft, or the stream. This includes overspraying of the site. Secondly, it can reach the site indirectly. In this case, spray drift is deposited on a non-target area (i.e. a road or farm track), and is washed off to the site during the next rain event. We defined spray drift to be possible if the application occurred within less than 100 m from the site (direct spray drift), or from a road or farm track draining to the site (indirect spray drift). Concentrations in this category may originate from spray drift or a process mentioned in the previous categories.
- *D) Surface runoff/(tile drainage)/spray drift/other.* This category was assigned if the pesticide was applied before the event and surface runoff to the sampling site was possible. This was defined to be the case if the application occurred within the surface runoff contributing area of the site (determination — see the “Surface runoff connectivity” section). Concentrations in this category may originate from surface runoff or processes mentioned in the previous categories. For the sites CS and ST, concentrations in this category may also originate from tile drainages.

Although it would have been desirable to further disaggregate the above-mentioned categories (e.g. surface runoff is a possible pathway, but spray drift is not), the spatio-temporal patterns in the study catchment did not allow for such a disaggregation. For example, there were no applications with the potential for surface runoff to a sampling location, but without spray drift potential.

As mentioned previously, for 4% of the agricultural area, no application data could be obtained. Since all concerned fields were situated far away from the sampling sites, the influence of the missing application data on our results can be neglected.

#### Discharge transported through inlets

As mentioned in the “Installations” Section 2.2.2, discharge in the inlets was calculated using water level measurements and a weir with a calibrated rating curve. The rating curve could only be calibrated for water levels corresponding to discharges of up to approximately 0.5 L/s. For higher water levels, we therefore calculated a minimum (*Q*_min_), a moderate (*Q*_mod_) and a high (*Q*_high_) discharge estimate (details in Section A1.3.2). For the discharge measured in the stream *Q*_stream_, no information on uncertainty was provided by the cantonal authorities. Expecting that the relative uncertainty of the discharge through inlets is much larger than the uncertainty in stream discharge, we neglected the latter.

To compare the discharge in the inlets and the stream, we calculated the ratio (*r*_Q,min_, *r*_Q.mod_, *r*_Q,high_) between the discharge estimate sums of all four inlets (*Q*_min_, *Q*_mod_, *Q*_high_) and the discharge in the stream (*Q*_stream_) (Eq. A3.1).Additionally, we calculated the ratio (*r*_Q,fast,min_, *r*_Q.fast,mod_, *r*_Q,fast,high_) between the discharge estimate sums of all four inlets (*Q*_min_, *Q*_mod_, *Q*_high_) and the fast discharge estimates in the stream (*Q*_stream,fast,high_, *Q*_stream,fast,mod_, *Q*_stream,fast,low_) (Eq. A3.2).

The fast discharge in the stream was estimated using a recursive filter technique (Lyne and Hollick, 1979) for discharge separation (function “BaseflowSeparation” of the R package “EcoHydRology”, version 0.4.12.1, Fuka et al. (2018)). We used three different filter parameters (0.9, 0.925 and 0.95; see Nathan and Mcmahon (1990)) to come up with a low, moderate and high estimate of the fast discharge.

Using the discharge measurements in the four inlets, we estimated the total discharge flowing through all inlets in the catchment *Q*_inl,tot_. For this, we used three simple extrapolation methods. In the first two methods, we assumed that the discharge in an inlet is proportional to the road area (Eq. A4.1) or the agricultural area connected to the inlet (Eq. A4.2). In the third method, we assumed that the discharge is proportional to the number of inlets (Eq. A4.3). These three methods are meant to provide a rough estimate of the total discharge, and other parameters influencing the total discharge (such as slope, soil permeability, crop types, spatial distribution of rainfall) were not taken into account.

#### Pesticide loads transported through inlets

To compare pesticide transport in the sampled inlets and the stream, we calculated pesticide loads and their ratio between the inlets and the stream. These calculations were only performed for events with sufficient temporal sampling resolution in the stream, i.e. events 5, 6 and 12, but not events with backup samples from cantonal authorities (see the “Field work” section). These three events correspond to the highest, fourth highest and sixth highest of the 19 rain events sampled with respect to pesticide concentration sums measured in the inlets. To account for the uncertainty in discharge measurements and for the uncertainty introduced by the analytical limits of quantification (LOQ), we calculated minimum, moderate and high estimates of the pesticide loads *f* (Eq. 1).

1 $${{\varvec{f}}}_{{\varvec{i}},{\varvec{e}},{\varvec{s}}}=\left(\begin{array}{c}{f}_{i,e,s,\mathrm{min}}\\ {f}_{i,e,s,\mathrm{mod}}\\ {f}_{i,e,s,\mathrm{high}}\end{array}\right)=\left(\begin{array}{c}{Q}_{i,e,\mathrm{min}}\\ {Q}_{i,e,\mathrm{mod}}\\ {Q}_{i,e,\mathrm{high}}\end{array}\right)\bullet \left(\begin{array}{c}{c}_{i,e,s,\mathrm{min}}\\ {c}_{i,e,s,\mathrm{min}}\\ {c}_{i,e,s,\mathrm{max}}\end{array}\right)$$

with: $${c}_{i,e,s,\mathrm{min}}=\left\{\begin{array}{c}{c}_{i,e,s} \left|{c}_{i,e,s}\ge {\mathrm{LOQ}}_{\mathrm{s}}\right.\\ 0 \left|{c}_{i,e,s}<{\mathrm{LOQ}}_{\mathrm{s}}\right.\end{array}\right.$$

$${c}_{i,e,s,\mathrm{max}}=\left\{\begin{array}{c}{c}_{i,e,s} \left|{c}_{i,e,s}\ge {LOQ}_{\mathrm{s}}\right.\\ {LOQ}_{s} \left|{c}_{i,e,s}<{\mathrm{LOQ}}_{\mathrm{s}}\right.\end{array}\right.$$

*f*_*i,e,s,min,*_* f*_*i,e,s,mod, *_* f*_*i,e,s,high*_: load estimates (ng) of substance s during event e at location i

*Q*_*i,e,min,*_*Q*_*i,e,mod,*_* Q*_*i,e,high*_: estimates of the total discharge (L)

*c*_*i,e,s,min,*_* c*_*i,e,s,max*_: minimal and maximal concentration of substance s (ng/L)

*LOQ*_*s*_: limit of quantification of substance s (ng/L)

From these estimates, we calculated the ratio between the loads measured in the four inlets and in the stream *r*_*f*_ for each substance and event (Eq. 2).

2 $${{\varvec{r}}}_{{\varvec{f}},{\varvec{e}},{\varvec{s}}}=\left(\begin{array}{c}{r}_{f,e,s,\mathrm{min}}\\ {r}_{f,e,s,\mathrm{mod}}\\ {r}_{f,e,s,\mathrm{high}}\end{array}\right)=\left(\begin{array}{c}\frac{\sum_{i=1}^{4}{f}_{inl,i,e,s,\mathrm{min}}}{{f}_{\mathrm{stream},e,s,\mathrm{high}}}\\ \frac{\sum_{i=1}^{4}{f}_{inl,i,e,s,\mathrm{mod}}}{{f}_{\mathrm{stream},e,s,\mathrm{mod}}}\\ \frac{\sum_{i=1}^{4}{f}_{inl,i,e,s,\mathrm{high}}}{{f}_{\mathrm{stream},e,s,\mathrm{min}}}\end{array}\right)$$

*r*_*f,e,s*_: load ratio estimates between inlets and the stream (-)

In a next step, we calculated the average of the minimal, moderate and high load ratios between the inlets and the stream using two different approaches. In the first approach, we calculated the mean of the load ratios of each single substance and event (*r*_*f,μ*,subst_; Eq. 3.1). In the second approach, we calculated the ratio between the substance load sums in the four inlets and in the stream (*r*_*f,μ*,sum_; Eq. 3.2).

3.1 $${{\varvec{r}}}_{{\varvec{f}},{\varvec{\upmu}},\mathbf{s}\mathbf{u}\mathbf{b}\mathbf{s}\mathbf{t}}=\frac{\sum_{s=1}^{{n}_{s}}\sum_{e=1}^{{n}_{e}}{{\varvec{r}}}_{{\varvec{f}},{\varvec{e}},{\varvec{s}}}}{{n}_{e}\bullet {n}_{s}}$$

3.2 $${{\varvec{r}}}_{{\varvec{f}},{\varvec{\upmu}},\mathbf{s}\mathbf{u}\mathbf{m}}=\frac{\sum_{s=1}^{{n}_{s}}\sum_{e=1}^{{n}_{e}}\sum_{i=1}^{4}{{\varvec{f}}}_{{\varvec{i}}{\varvec{n}}{\varvec{l}},{\varvec{i}},{\varvec{e}},{\varvec{s}}}}{\sum_{s=1}^{{n}_{s}}\sum_{e=1}^{{n}_{e}}{{\varvec{f}}}_{\mathbf{s}\mathbf{t}\mathbf{r}\mathbf{e}\mathbf{a}\mathbf{m},{\varvec{e}},{\varvec{s}}}}$$

*n*_*s*_: number of substances s measured (-)

*n*_*e*_: number of events e sampled (-)

In a last step, we used the same extrapolation approach as for the discharge (“Discharge transported through inlets” section) to come up with a rough estimate of the pesticide load ratio between all inlets in the catchment and the stream.

#### Model of concentrations in inlets

To better understand which factors influence the pesticide concentrations in inlets, we created a linear mixed model with the measured inlet concentrations log_10_(c) as a response variable (function “lmer” of the R package “lme4”, version 1.1.27.1, Bates et al. (2015)). As potential explanatory variables, we chose a set of variables commonly considered important for pesticide transport: time since application *t*_appl_, amount of substance applied log_10_(*m*_appl_), Freundlich adsorption coefficient normalised to organic carbon content log_10_(*K*_foc_), octanol–water partition coefficient log_10_(*K*_ow_), substance half-life in water DT_50,water_, substance half-life in soil DT_50,soil_, moderate estimate of the discharge in the inlet during the event log_10_(*Q*_mod_), type of potential transport processes involved *p*_transport_ (see the “Transport processes” section) and the inlet sampled *i* (details in Table A2). Substance properties were obtained from Lewis et al. (2016). The inlet sampled *i* was defined as a random factor, all other variables as fixed variables. Since the variables log_10_(K_foc_) and log_10_(K_ow_) were strongly correlated, log_10_(K_ow_) (i.e. the variable with the lower AIC criterion resulting from single variable deletions) was removed. For the analysis, the dataset was reduced to those 20 substances with substance properties available and at least one application in the contributing area of an inlet (details in Table A3).

## Results and discussion

### Surface runoff connectivity

**Fig. 3:**
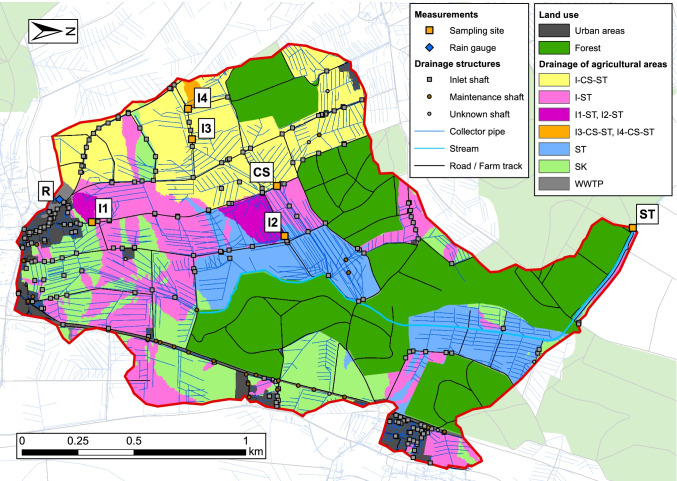
Surface runoff connectivity of the catchment. Yellow squares show the sampling sites (I1–I4: inlets; CS: collector shaft; ST: stream) and the rain gauge (R) is indicated by a blue diamond. Coloured areas show the contributing areas (CAs) of the inlets, sinks, and the stream. I-CS-ST: CAs of inlets draining through the collector shaft into the stream (these inlets were not sampled). I-ST: CAs of inlets draining to the stream without passing the collector shaft (these inlets were not sampled). I1-ST, I2-ST: CAs of inlets 1 and 2, draining to the stream without passing the collector shaft. I3-CS-ST, I4-CS-ST: CAs of inlets 3 and 4, draining through the collector shaft to the stream. (The CA of inlet 3 is small and therefore not visible on the map.) ST: areas directly drained to the stream. SK: areas draining to a sink. WWTP: areas drained to a wastewater treatment plant

The results of the surface runoff connectivity model (Fig. 3) show that around 76% of the agricultural area in the catchment has a surface runoff connectivity to the stream. From this area, 25% is directly connected to the stream, and 75% is indirectly connected via inlets. The four sampled inlets drain around 5.7% of the agricultural area connected to inlets in the study catchment and 2.9% of the roads connected to inlets. The collector shaft drains around half of the agricultural and road area in the catchment that is connected to inlets. The remaining agricultural area (24%) is connected to sink areas. Although the water flowing into these sinks is expected to infiltrate, there might still be a connectivity to the stream via subsurface processes, such as tile drainage or ground water flow.

These findings are robust when considering the parameter uncertainty of the topographical model. The median area fractions connected to the stream resulting from the Monte Carlo simulation corresponded to 73% of the agricultural areas and the indirect connectivity dominates (83% of the connected agricultural area, or 61% of all agricultural areas). These simulations also allowed us to compare the connectivity of the study catchment to a national connectivity assessment (Schönenberger and Stamm, 2021). The comparison revealed that the study catchment represents conditions with a very high fraction of indirectly connected agricultural area (97% quantile of the national distribution). The median of the national distribution (35%) is approximately 1.7 times lower than that in the study catchment (61%). Accordingly, we expect that in an average Swiss arable land catchment, surface runoff via inlets and related pesticide transport is lower than in the study catchment, but in a similar order of magnitude. Source of background map: Swisstopo (2020).

### Hydrological behaviour of inlets

During the study period, 37 rain events were recorded. Their duration was between 1 and 41 h (median: 9 h). During 34 rain events, discharge was measured in at least one of the inlets (see Figure A16). The discharge formation in the inlets depended on the total rainfall sum of the respective rain event, but not on the rainfall intensity. The rainfall needed to trigger discharge differed between the inlets. The minimal rainfall sum needed was 1.3–1.5 mm for I1, I2 and I4, while I3 was only getting active with 3.6 mm (details in Table A4). This can be explained by the grass strip separating I3 from the adjacent road (see the “Sampling site selection” section). Additionally, due to the seepage through the shaft bottom of I3 during dry periods, surface runoff entering the inlet first had to fill the shaft, before being transported through the outlet pipe. Similarly, the measured discharge differed strongly between the four inlets, being much higher in I1 and I2 than in I3 and I4 (details in Figure A15).

**Fig. 4:**
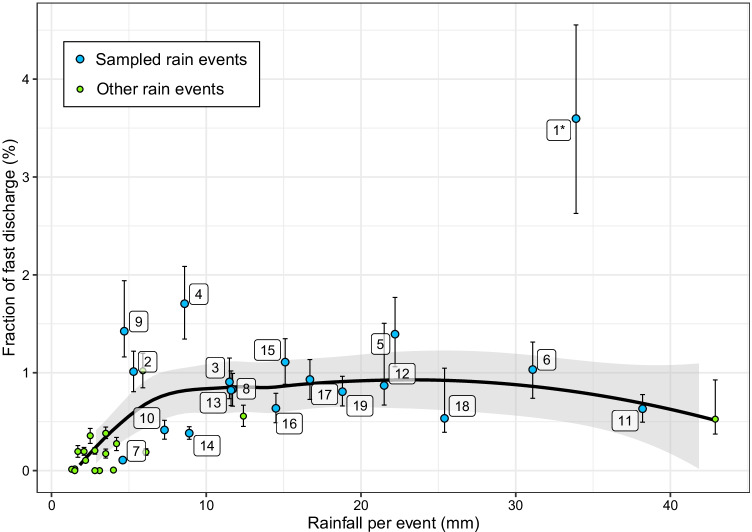
Ratio between the discharge sum in the four inlets and the fast discharge in the stream *r*_Q,fast_. Points correspond to the moderate estimates (*r*_Q,fast,mod_), error bars to the minimum and high estimates (*r*_Q,fast,min_ and *r*_Q,fast,high_). Sampling event numbers are indicated with white boxes. The numbers represent the events in ascending order of time. The black line represents a smoothed conditional mean of *r*_Q,fast,mod_, obtained by a locally weighted scatterplot smoothing (LOESS) using the R package ggplot2 (version 3.3.3, function geom_smooth). The grey area represents the corresponding 95% confidence interval. Event 1 was a snowmelt event and was therefore excluded from the analysis

For each rain event, the ratio between the discharge sum of all four inlets and the fast discharge fraction in the stream (*r*_Q,fast_) is shown in Fig. 4. For small events (rainfall < 4 mm), the four inlets are only responsible for less than 0.4% of the fast discharge in the stream. For larger events (rainfall > 10 mm), the contribution is higher with an average 0.83% (0.64 to 1.1%; see Table A5). Event 1 is a clear outlier with *r*_Q,fast_ equalling around 3.6%. During this event, the ground was covered by melting snow. The snow on the farm tracks was melting faster than that on the agricultural areas, explaining the higher discharge transported through the inlets. For small events, the estimation of fast discharge based on discharge separation underlies large uncertainties and should be interpreted with care. A comparison of the discharge sum of all four inlets to the total discharge in the stream (*r*_Q_) revealed similar results with higher contributions of inlets for rain events > 10 mm (details in Figure A17).

The results of the discharge extrapolation from the measured inlets to all inlets in the catchment indicate rain events larger than 10 mm, between 3.6 and 10% of the total discharge and between 11 and 43% of the fast discharge in the stream originates from inlets (details in Table A5). These numbers are lower than it would be expected from the connectivity analysis, which estimated that 75% of the areas with surface runoff connectivity are connected to the stream via inlets. This indicates that the fast discharge in the stream originated to large amounts from other sources than direct and indirect surface runoff from agricultural roads or fields. We hypothesise that preferential flow through tile drainages, surface runoff formed on urban areas, or the fast outflow of pre-event water were major other sources of fast discharge in the stream.

The measurements and extrapolations reported above are only based on measurements in four out of 158 inlets in the catchment. Obviously, the extrapolation to the entire catchment can only provide a very rough estimate of the overall relevance of inlets on the catchment hydrology. In addition, our discharge measurements were restricted to inlets along farm tracks, being the most frequent inlet type in the catchment. Inlets along concrete roads are, however, expected to react much faster (i.e. produce runoff at lower rainfall sums) and to show higher runoff coefficients. In contrast, inlets located directly in fields are expected to react slower and to show lower runoff coefficients. On a national scale, most inlets are located along concrete roads (Schönenberger and Stamm, 2021). We therefore expect that in most other catchments, inlets tend to react faster and to have higher runoff coefficients.

### Concentrations and loads

#### Measured concentrations and loads

Inlet water samples were analysed for 19 of 37 rain events, covering 80% of the total discharge transported through the sampled inlets during the study period. In the remaining events, either discharge was too small to trigger sampling (15 events), or no sampling bottles were installed (3 events). Additionally, for six of these events, water samples from the collector shaft and the stream were analysed (details in Table A6). From the 51 substances measured, 43 were found in at least one sample. Between 22 and 33 substances were found in the inlets and the collector shaft, and 42 in the stream (Table 1). The measured concentrations differed strongly between sampling sites. The highest pesticide concentrations were found in I4 for both, mean (291–322 ng/L) and maximal (62,000 ng/L, terbuthylazine) concentrations. However, high pesticide concentrations were also found in I1, the collector shaft and the stream. In contrast, pesticide concentrations in I2 and I3 were much lower. A table with all measured concentrations is provided in SI-B.

**Table 1:** Overview over the pesticide concentrations measured at the different sampling sites. Due to the uncertainty caused by the limit of quantification (LOQ), a range is provided for the mean concentrations. For calculating the lower limit of this range, we replaced the concentrations below the LOQ by 0. For calculating the upper limit, we replaced them by the LOQ. An overview over the measured transformation product concentrations are provided in Table A7. I1–I4: inlets; CS: collector shaft; ST: stream

Site	I1	I2	I3	I4	CS	ST
Number of substances above LOQ	33	26	22	25	33	42
Mean pesticide concentration (ng/L)	92–124	9–40	11–43	291–322	51–65	190–201
Maximal pesticide concentration (ng/L)	7900	920	500	62,000	7900	35,000
Pesticide with highest concentration	Metamitron	Metamitron	Diflufenican	Terbuthylazine	Terbuthylazine	Propamocarb

The sampling procedure in the inlets (water-level proportional) was different from the one in the collector shaft and the stream (time proportional) which can introduce a bias in the measured concentrations (Schleppi et al., 2006; Liger et al., 2012; Bundschuh et al., 2014). Moreover, the number of events analysed differed between these sites. Therefore, a direct comparison of the concentrations in the inlets to the collector shaft or the stream should be performed with caution. Load calculations — as presented and discussed in the “Relevance of inlets at the catchment scale” section — are more appropriate for a comparison.

In contrast, the concentrations of water-level proportional composite samples in inlets can be compared directly. However, during some events, composite samples were not taken in some inlets, mostly due to lack of sufficient surface runoff (see above). Instead, grab samples from the stagnating water were taken after the event (details in Table A6). Rübel (1999) showed that the pesticide concentrations in surface runoff from vineyard roads are approximately constant within a rain event and that the mixing of different water sources caused the concentration variations observed in the stream. Assuming that this also holds for roads around arable crops, grab sample concentrations can be compared directly to the concentrations of water-level proportional samples, as it is done in the following.

**Fig. 5:**
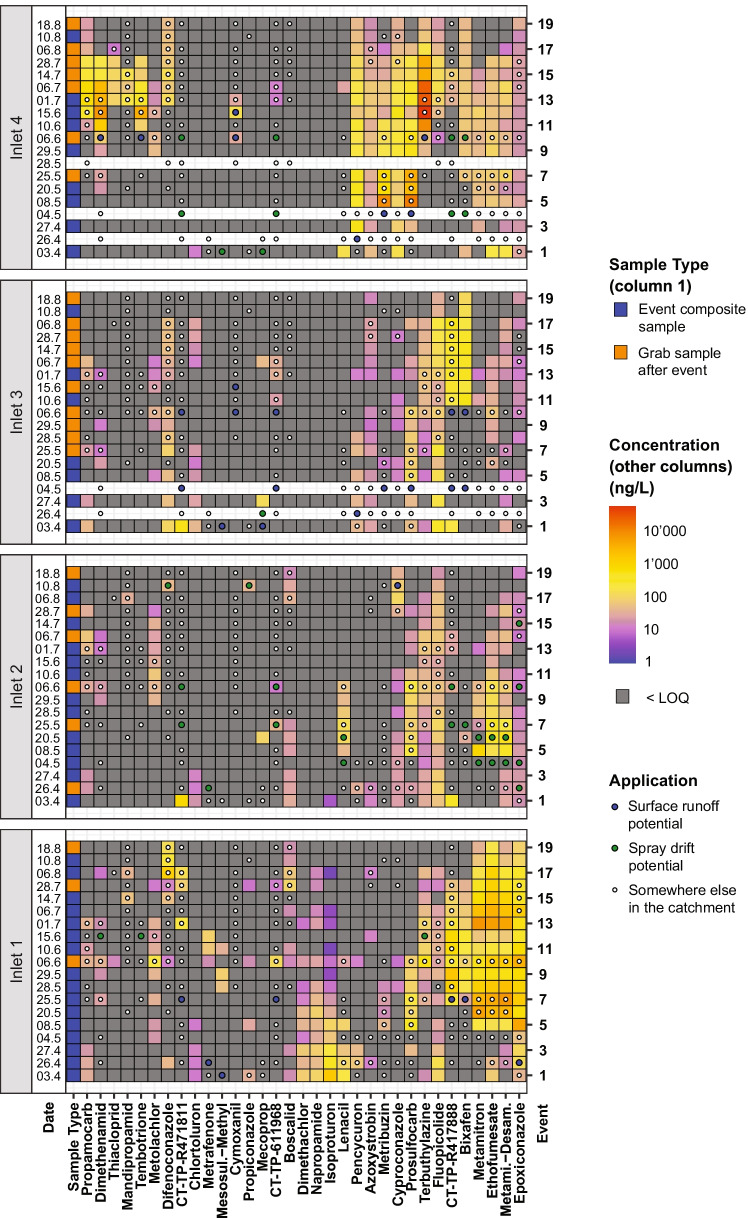
Concentrations c (ng/L) measured in inlets for events 1 (3 April 2019) to 19 (18 August 2019). Only substances found at least twice in concentrations > 25 ng/L are shown. White rows indicate that no sample was taken. In the first column, the sample type is indicated. In the remaining columns, substances are clustered by the concentrations measured. Coloured dots indicate that the particular substance was applied in the period between the respective and the previous event. Dot colours specify the potential transport processes

The temporal concentration patterns in the inlets differed strongly between pesticides (Fig. 5). Many substances were persistently measured over periods of 2 months or longer (e.g. metamitron and epoxiconazole at I1, penycuron and metribuzin at I4). This especially holds for substances found in high concentrations. However, other substances were only found in a single sample or two consecutive samples (e.g. propiconazole, cymoxanil or mecoprop). How these patterns align with pesticide applications and properties is presented in the “Factors influencing pesticide concentrations in inlets” section.

Similarly, also the measured loads varied strongly between the inlets and in time. I1 was responsible for the largest fraction of the total load per pesticide transported through the sampled inlets (45%), followed by I4 (30%), I2 (19%) and I3 (6%) (details in Figure A24). Further details on the transported loads are provided in the “Relevance of inlets at the catchment scale” section.

#### Factors influencing pesticide concentrations in inlets

##### Transport processes

We combined the pesticide application data (time, location, substance and amount applied) with the temporal evolution of the concentrations in the inlets. Based on these datasets, we were able to allocate potential transport processes to each measured concentration. This allocation was based on the spatio-temporal relationship between the application and the measured sample, as described in the “Transport processes” section. It allowed gaining insights on the relevance of the different transport processes and other influencing factors on pesticide concentrations in inlets. In Fig. 5, the temporal development of the concentrations of the most important compounds is depicted for the 19 sampling events (see Figure A18 and Figure A19 for similar plots for all compounds and the sites CS and ST). Additionally, the respective application timing and potential related transport processes (surface runoff, spray drift, other) are provided. A disaggregated version of this plot with a continuous time axis and including precipitation is provided in the supplementary information on the example of epoxiconazole at I1 (Figure A21) and pencycuron at I4 (Figure A22).

**Fig. 6:**
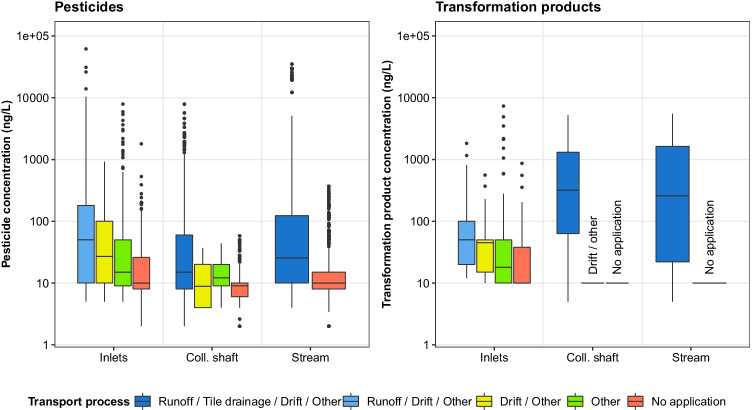
Distribution of pesticide and transformation product concentrations for the sampled inlets, the collector shaft and the stream. Concentrations are assigned to possible responsible transport processes. For substances below the limit of quantification (LOQ), the LOQ was used for the analysis. A more detailed version of this plot, showing each inlet separately, is provided in Figure A23

These data reveal that applications on fields with surface runoff or spray drift potential to inlets led to strong concentration increases in the corresponding inlets. This was usually observed during the first three events after the application (e.g. bixafen at I1 and I3, terbuthylazine at I4). The highest concentration measured in inlets (terbuthylazine at I4) was related to such an application. Although such a response was not observed in all cases (e.g. metrafenone at I2, cymoxanil at I3), median concentrations in the inlets were clearly related to the potential transport processes (Fig. 6). The median concentrations in the inlets decreased from potential surface runoff (category D) over potential spray drift (category C) to other transport processes related to pesticide applications in the catchment (category B), and finally other transport processes not related to a pesticide application in the catchment (category A). This pattern was not only found for pesticides, but also for transformation products. A similar concentration decrease between transport process categories was found in the collector shaft and in the stream.

In summary, high pesticide concentrations in the inlets can be explained in many cases by prior applications on fields with surface runoff or spray drift potential to the corresponding inlet. However, also applications on fields without the potential for these processes to occur led to high concentrations in inlets of up to 7900 ng/L (e.g. metamitron and ethofumesate at I1, propamocarb at I4). The same holds for substances with no application at all reported in the catchment before the respective event (e.g. napropamide and isoproturon at I1, chlortoluron at I1–I4; maximal concentrations up to 1800 ng/L). These results show that also other mechanisms besides surface runoff and spray drift were responsible for high concentrations in inlets. These mechanisms may involve droplet losses, accidental spills, residual wash off from applications in previous years, unreported applications, applications outside the study catchment, or (only in case of I1) farmyard runoff.

The highest concentrations related to applications on fields without surface runoff or spray drift potential were measured in I1 (metamitron and ethofumesate). By rechecking with the farmers, we could exclude unreported applications to be responsible for these concentrations. Additionally, metamitron and ethofumesate have a rather fast degradability (DT_50,soil_: 19 and 22 days; DT_50,water_: 11 and 20 days) and were not applied in the contributing area of the inlet in the year before this study, speaking against wash off of residuals as a source. However, I1 is located close to a village at a farm track often used by farmers for accessing their fields in or outside the study catchment. In contrast, the other inlets are located along farm tracks less often used. This indicates that droplet losses from leaking spraying equipment or accidental spills on the farm track could be responsible for the increased concentrations in I1.

Also in the other inlets, certain substances with rather high degradability (DT_50_,_soil_ < 25 days) were found in elevated concentrations > 100 ng/L without related applications with surface runoff or spray drift potential (e.g. prosulfocarb at I2, ethofumesate at I4). This again indicates that for some substances droplet losses or accidental spills (but potentially also unreported applications) are responsible for high concentrations in inlets. Contrarily, also substances with low degradability (DT_50_,_soil_ > 270 days) were measured in elevated concentrations in inlet samples without related applications with surface runoff or spray drift potential (e.g. fluopicolide at all inlets, napropamide at I1). These concentrations likely originated from residual wash off from applications in previous years.

In summary, high pesticide concentrations in inlets are mainly caused by the following transport processes: applications with the potential for surface runoff or spray drift, and potentially droplet losses from leaking spraying equipment or accidental spills on the farm track. This aligns well with studies performed for surface waters, where the same processes have been shown to cause high pesticide concentrations (Holvoet et al., 2007; Reichenberger et al., 2007).

##### Other influencing factors

The influence of transport processes on the pesticide concentrations in inlets is also shown in the results of the linear mixed model. From all variables tested, the strongest effects on concentrations were observed for the potential transport processes *p*_transport_.

However, also other factors strongly influenced pesticide concentrations in inlets (details in Table A8). For substances applied on fields with surface runoff or spray drift potential to inlets, high concentrations in the inlets were significantly related to substances with low degradability (DT_50,soil_: *p* < 0.001, DT_50,water_: *p* < 0.005). Such persistent substances are commonly found in streams during dry weather (Kreuger, 1998; Hermosin et al., 2013; Halbach et al., 2021) and can be explained by delayed tile drainage or ground water flow (Reichenberger et al., 2007; Gramlich et al., 2018). However, even though tile drainage and ground water flow cannot enter the inlets, substantial single pesticide concentrations (up to 26,000 ng/L) were found in the grab samples taken in the inlets after the events (Fig. 5). This shows that the stagnating water in the inlets (and possibly eroded soil deposited at the inlet bottoms) acted as a pesticide reservoir. Consequently, after an initial rain event with pesticide input, inlets act as pesticide sources and may even lead to pesticide transport to surface waters during rain events with clean surface runoff. This reservoir effect has previously been shown for natural stagnant water bodies (Ulrich et al., 2021), but also to a lesser extent (much lower concentrations) for constructed wetlands (Maillard and Imfeld, 2014; Imfeld et al., 2021). Constructed wetlands are usually reported to overall reduce pesticide transport to surface waters and are therefore often used as a mitigation measure (Vymazal and Březinová, 2015). It was shown that their capability to retain pesticides increases with their density of plant coverage and their hydraulic retention time (Stehle et al., 2011). Inlets have no plant coverage and only a very short hydraulic retention time. Therefore, if we assume that inlets are a special type of constructed wetland, we expect that their efficacy in reducing pesticide transport to surface waters is low and that they act as a pesticide reservoir instead. This aligns well with the results presented here.

Also the Freundlich adsorption coefficient normalised to the organic carbon content log_10_(K_foc_), the amount of substance applied log_10_(m_appl_) and the time since application t_appl_ were found to significantly influence the concentrations in the inlets (see Table A8). The Freundlich adsorption coefficient and the time since application were correlated negatively to the concentrations in the inlets, while the amount of substance applied was correlated positively. These variables have been previously reported to be important influencing factors for pesticide transport to surface waters (Reichenberger et al., 2007; Boithias et al., 2014). Consequently, our results indicate that pesticide transport to inlets and to surface waters is affected by the same substance properties.

In contrast to the above-mentioned factors, the discharge transported through the inlets per event did not appear as a significant influencing factor in the model. This aligns well with a study by Imfeld et al. (2020) reporting that the event concentrations at the outlet of a small vineyard catchment were related to the timing of pesticide applications, but not to characteristics of the rain events.

#### Relevance of inlets at the catchment scale

##### Relevance of sampled inlets

**Fig. 7:**
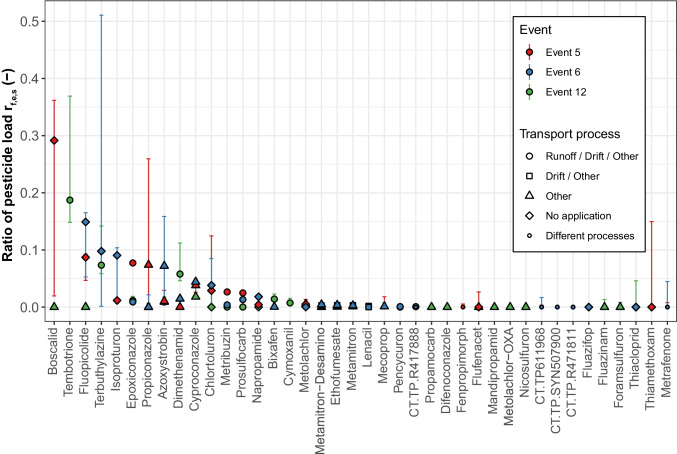
Ratios between the sum of pesticide loads transported through the four sampled inlets and the stream (*r*_f,e,s_) during selected events (events 5, 6 and 12). Dots represent the moderate estimates (*r*_f,e,s,mod_), and error bars the minimum (*r*_f,e,s,min_) and high (*r*_f,e,s,high_) estimates. Different dot types represent the transport process categories supposed to cause transport to the sampled inlets

In agreement with the large spatio-temporal variability of pesticide concentrations and loads in the sampled inlets, also their contribution to the overall load in the stream largely differed. This is illustrated by Fig. 7, showing the load ratios of each pesticide between the sampled inlets and the stream (*r*_f_) for selected events. In some situations, transport through these inlets contributed considerably to the total load of certain pesticides in the stream: In four cases, 10% or more of the load originated from the sampled inlets. In three of these cases, this load was even caused by a single inlet only. However, 40 out of 93 cases, the sampled inlets were of negligible importance for the load in the stream. Overall, the average load ratio per substance between the sampled inlets and the stream (*r*_f,μ,subst_) was approximately 1.8% (0.8 to 3.7%) (details in Table A9). In contrast, the ratio between the load sums of all substances in the inlets and in the stream (*r*_f,μ,sum_) equalled approximately 0.3% (0.2 to 0.5%). The difference between these two ratios can be explained by few single substances contributing to large extents to the total load in the stream. For example, in event 12, propamocarb alone was responsible for 56% of the total load in the stream.

The differences between the maximum and minimum estimates of *r*_f,μ,subst_ and *r*_f,μ,sum_ to their moderate estimates were mainly caused by the analytical LOQ. This analytical uncertainty is responsible for 75% and 92% of the total difference between maximum and minimum estimates to the moderate estimates. The remaining differences are caused by the discharge measurement uncertainty. For reducing the load uncertainty in further studies, the focus should therefore be rather set on using analytical methods with lower LOQs than on improving the accuracy of discharge measurements.

Pesticide load ratios (*r*_f_) were not related to a specific type of potential transport process to the inlets. High pesticide load ratios were found for all transport process types and even for substances without recorded applications (Fig. 7). However, high absolute loads (*f*) were in most cases related to applications in the study catchment (Figure A25). From 46 cases with loads of more than 1 mg in inlets, 20 each were related to a pesticide application with surface runoff potential, and potential for other transport processes only. From the remaining cases, three were related to an application with spray drift potential, and three to either residual wash off from applications in previous years, unreported applications or applications outside the catchment.

The load ratios reported above were only determined for three rainfall events with rather high pesticide concentration sums measured in the inlets compared to the other events of the study period (see the “Pesticide loads transported through inlets” section). Likely, the load ratios are therefore smaller for the remaining events.

Besides discharge uncertainty and analytical uncertainty (see above), the different types of sampling methods used (time-proportional in the stream, water-level proportional in inlets) are an additional source of uncertainty in the load calculations. For both methods, the uncertainty related to the sampling method may be substantial if the temporal variations of discharge and concentrations are large within the period covered by a mixed sample. In the stream, however, the temporal sampling resolution was high (see Figure A20 for an example). Therefore, the variation of discharge and concentrations per mixed sample is rather small. Accordingly, we also expect the stream load uncertainty caused by the sampling method to be rather small. For water-level proportional sampling, the influence of temporal variations of discharge and concentrations on the load uncertainty is generally smaller than that for time proportional sampling due to the correlation of water level and discharge. As mentioned previously, Rübel (1999) showed that the variation of concentrations on vineyard roads was small during single rain events and stated that a single sample per event is able to represent the event concentration well. Assuming that this conclusion can be transferred to roads around arable crops, we therefore also expect the water-level proportional sampling method to have a small influence on the uncertainty in load calculations.

##### Relevance of all inlets in the catchment

Based on the load ratios calculated for the sampled inlets and the contributing area characteristics of all inlets, we extrapolated the loads to the entire catchment. We estimate that during the selected events, on average around 30 to 70% of the load of each substance in the stream *r*_f,μ,subst_ originated from an inlet in the catchment (details in Table A9). With regard to the load sum ratio *r*_f,μ,sum_, we estimate that inlets were responsible for around 5 to 12%.

As already mentioned for the discharge extrapolation, this estimation is only based on measurements in four out of 158 inlets in the catchment. However, substantial differences were found between the loads transported through the four inlets. We therefore suppose that the selection of sampled inlets strongly influenced the load ratios calculated on the catchment scale. For a more robust estimate, additional measurements in other inlets would be essential. Moreover, additional measurements could help to create a more elaborate extrapolation model and to further improve the catchment scale load estimation.

Despite these uncertainties, our results indicate that — at least during some rain events — surface runoff transported through inlets in our study catchment contributed to substantial amounts to the total pesticide load in the stream. Our results are in line with the only other study reporting load ratios for agrochemicals transported through inlets (Remund et al., 2021). In this study, 88% of sediment and phosphorus losses to surface waters occurred through inlets or maintenance shafts. In other countries, storm drainage of fields and adjacent roads is often established by roadside ditches or the roads themselves. In accordance with our results, high pesticide concentrations have been measured in such roadside ditches (Rübel, 1999). Furthermore, in a small agricultural catchment, Louchart et al. (2001) reported that the fast transport of surface runoff via roadside ditches was responsible for 83% and more of the load of two herbicides lost to the stream. In a different catchment, a similar effect was reported for transport via roads (Lefrancq et al., 2014). These results corroborate that structures establishing a surface runoff connectivity between fields and surface waters generally entail a large risk for the transport of substantial pesticide loads to surface waters.

### Implications for other catchments

This study was performed in a single catchment and for four inlets only. In the following, we will elaborate which results are rather case-specific and which results can be extrapolated to other catchments.

We found that pesticide concentrations in single inlets can be very high, and that single inlets can be responsible for a large fraction of the pesticide load found in the stream. Assuming that the same processes are driving pesticide transport in other catchments, we suspect that high pesticide transport through inlets may potentially occur in every catchment in which inlets exist and pesticides are applied. Whether high pesticide concentrations and loads effectively occur in a given inlet depends on a complex interaction of local influencing factors allowing the above-mentioned transport processes to happen. If pesticide transport is dominated by surface runoff and spray drift, important factors include the spatial arrangement of sprayed crops, roads and inlets, the local topographical conditions, rainfall patterns, wind conditions, soil and crop types, soil management, type and amount of pesticide applied, and the type of spraying equipment. If pesticide transport is dominated by accidental spills and droplet losses, important factors are the care of farmers during pesticide application and the condition of the spraying equipment.

In the “Relevance of inlets at the catchment scale” section, we estimated the ratio of the pesticide load transported through all inlets in the whole catchment during three rain events. This estimation suggests that a very large ratio (30 to 70%) of the pesticide load measured in the stream was transported through inlets. It remains unclear if this ratio is smaller or larger for other catchments and rain events. In the following, we first discuss arguments supporting smaller ratios, and then arguments supporting larger ratios.

The load ratio reported above was calculated for three rain events with rather high pesticide concentrations in the inlets compared to the other events (see the “Measured concentrations and loads” section). During the other events, we therefore expect the average load ratio to be smaller. Furthermore, compared to an average agricultural catchment in Switzerland, a high fraction of the agricultural area (1.7 times higher than the median) is connected to the stream via inlets in our study catchment (see the “Surface runoff connectivity” section). Both considerations indicate that the load ratios reported here are rather case-specific and might on average be smaller for other catchments and rain events.

Contrarily, two different arguments indicate that the average load ratios transported through inlets could be higher in other catchments than the values reported here. First, as mentioned in the “Hydrological behaviour of inlets” section, our measurements were performed at inlets located along farm tracks. However, on the national level, most inlets are located along concrete roads. On concrete roads, surface runoff is formed already for very small rainfall events. Therefore, we suppose that on concrete roads, the time between pesticide applications and the next rain event causing surface runoff formation is smaller. This could lead to reduced degradation and to increased wash-off of spray drift deposited on roads compared to farm tracks. Second, as mentioned in the “Study catchment” section, the farmers in the catchment were participating in a programme aiming on the reduction of pesticide pollution in the receiving stream. They were aware that transport through inlets might lead to pollution of the stream and that pesticide concentrations are measured in inlets. Thus, especially around the sampled inlets, they were most probably more careful with pesticide handling than farmers in other catchments, leading to lower pesticide transport through the sampled inlets.

### Role of application data for process understanding

In many studies conducted on pesticide transport on the catchment scale, application data are not available at all, only in aggregated form, or with other limitations (Hunt et al., 2006; Zhan and Zhang, 2014). Full data sets are often difficult to obtain since the consent and cooperation of all farmers in the catchment is needed, and privacy protection has to be ensured. For this study, we received an almost full dataset of pesticide applications in the study catchment. Even though we were only allowed to report the application data in a aggregated form to ensure privacy protection, our study highlights that linking measured pesticide concentrations to transport processes is only possible given the simultaneous availability of sufficiently resolved application data (plot resolution, daily scale) and sampling data (single inlets, event scale). Without such data, we would have been unable to identify the importance of the different pesticide transport mechanisms in the study catchment or the relevance of compound properties. Moreover, we likely would have confused mechanisms of category B (other processes) with category C or D (surface runoff or spray drift). Consequently, studies aiming to improve the understanding of pesticide transport processes in agricultural catchments should put effort into simultaneously collecting application and sampling data of sufficient spatio-temporal resolution.

Nevertheless, our study also shows that even with available high-resolution application and sampling data, some of the pesticide transport processes had to be suspected (e.g. droplet losses or accidental spills on farm tracks). This illustrates that the pesticide transport processes in agricultural catchments are still poorly understood.

## Conclusions

In this study, discharge and pesticide concentrations were measured for the first time in inlets agricultural storm drainage systems. These inlets were shown to strongly influence surface runoff and related pesticide transport in the studied catchment: The concentrations of single pesticides in inlets amounted up to 62 μg/L and during some rain events, single inlets were responsible for more than 10% of the load of a certain pesticide in the stream. In a rough extrapolation, we estimated that inlets were responsible for 3.6 to 10% of the total discharge in the stream, and for 11 to 43% of the fast discharge fraction. For a subset of three selected large rain events, 30 to 70% of the average load per pesticide in the stream originated from inlets. These pesticide load ratios are however rather case-specific and it is difficult to say if the load ratios in other catchments are larger or smaller. To determine which ratio of pesticide pollution in streams originates from inlets, further studies in other catchments are therefore inevitable. Nevertheless, a comparison to other studies suggests that structures increasing the surface runoff connectivity from fields and adjacent roads to surface waters (e.g. inlets, roadside ditches, roads) generally entail a high risk for pesticide loads to surface waters.

This study also provided insights into the processes leading to increased concentrations in inlets. High concentrations were often related to recent pesticide applications on fields with surface runoff or spray drift potential to the sampled inlets. However, increased concentrations in inlets were also found in other cases. Our results indicate that droplet losses or accidental spills on farm tracks may have caused those increased pesticide concentrations. The amount of substance applied, the time since application and substance properties (DT_50soil_, DT_50water_, K_foc_) were identified as other variables with a significant influence on the pesticide concentrations in inlets.

In summary, we conclude from this study that pesticide transport through storm drainage inlets can be a relevant pathway for pesticide pollution of surface waters. This transport pathway should therefore receive more attention in future research, but also in pesticide registration and legislation, and during the application of pesticides.

## Supplementary Information

Below is the link to the electronic supplementary material.

Supplementary Information A (PDF 10.2 MB)

Supplementary Information B (XLSX 62.4 KB)

## Data Availability

The datasets generated and analysed during the current study (e.g. pesticide concentrations in all samples, rainfall data, discharge data) are available from the Eawag Research Data Institutional repository (10.25678/0005X4).
