# Supplementary material for: Pesticide concentrations in agricultural storm drainage inlets of a small Swiss catchment
Source: Environ Sci Pollut Res Int. 2022 Feb 5;29(29):43966–83. doi: 10.1007/s11356-022-18933-5 (PMC9200698; doi:10.1007/s11356-022-18933-5)
Supplement: Supplementary file 1 — Supplementary Information A (PDF 10.2 MB) [file 11356_2022_18933_MOESM1_ESM.pdf]

Supplementary Information A (SI-A) to:

Pesticide concentrations in agricultural storm drainage inlets of a small Swiss catchment

Urs T. Schönenberger[1,*], Birgit Beck[1], Anne Dax[1], Bernadette Vogler[1], and Christian Stamm[1]

[1]Eawag, Swiss Federal Institute of Aquatic Science and Technology, 8600 Dübendorf, Switzerland.

[*]Corresponding author: urs.schoenenberger@eawag.ch

The datasets generated and analysed in this study (e.g. pesticide concentrations, rainfall data, discharge data) can be downloaded from the Eawag Research Data repository (doi: 10.25678/0005X4).

Table of contents

A1. Methods **3**
A1.1 Field work 3
A1.1.1 Sampling sites 3
A1.1.2 Water-level proportional samplers 7
A1.1.3 Sampling strategy 9
A1.1.4 Field mapping 10
A1.2 Chemical analysis 10
A1.3 Data analysis 12
A1.3.1 Surface runoff connectivity model 12
A1.3.2 Discharge measurement in inlets 12
A1.3.3 Model of concentrations in inlets 16
A2. Results **20**
A2.1 Hydrological behaviour of inlets 20
A2.2 Concentrations and loads 23
References **30**

A1. Methods

A1.1 Field work

A1.1.1 Sampling sites

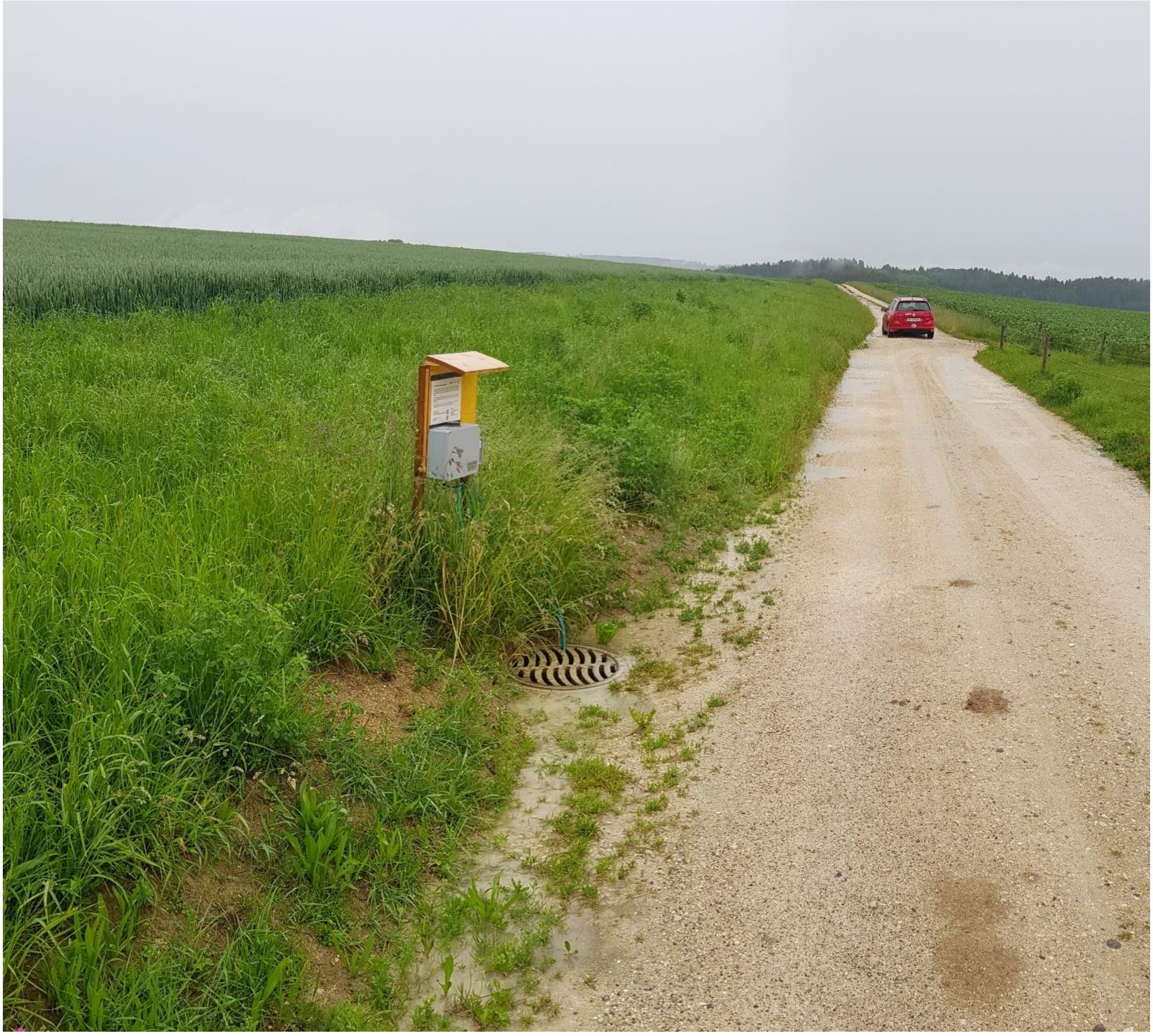

Figure A1: Sampling site I1. The inlet is situated between a rather flat farm track and a wheat field with moderate slope. The field is separated by a buffer strip of approximately 6 m width from the farm track.

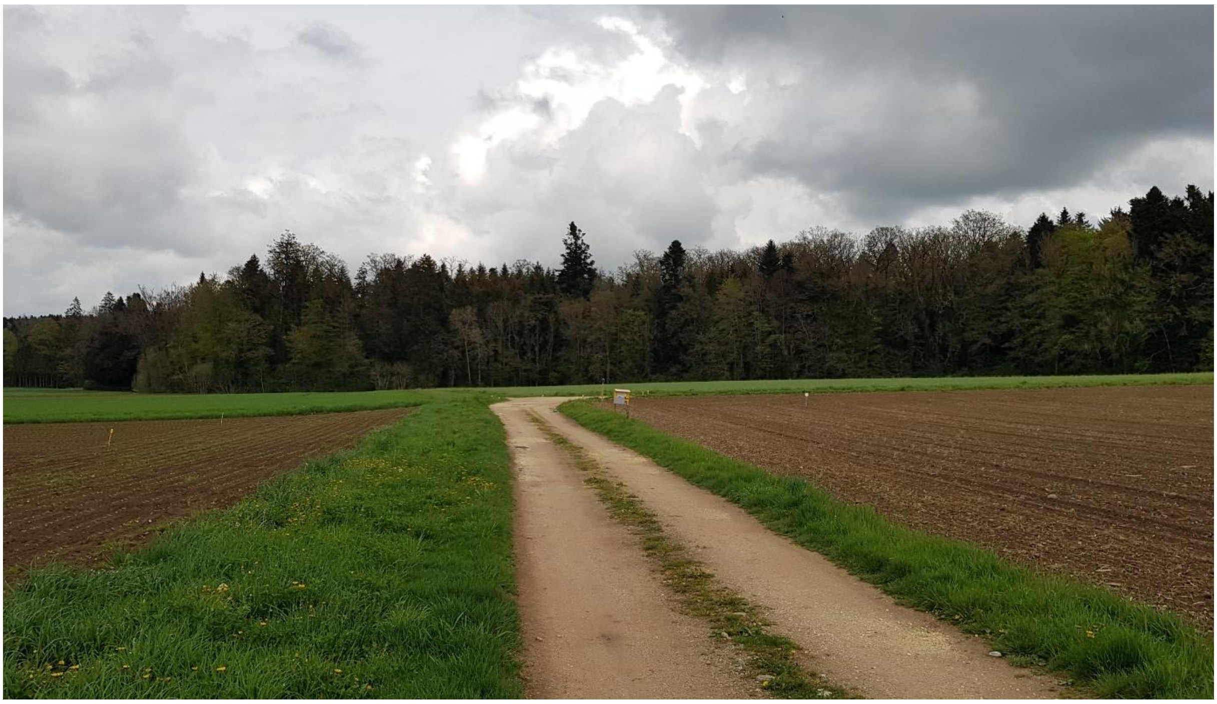

Figure A2: Sampling site I2. The inlet is situated between a flat farm track and a flat sugar beet field. The field is separated from the farm track with a buffer strip of approximately 0.5 m width. The inlet itself is located on the buffer strip and therefore lies directly at the border of the field and the farm track.

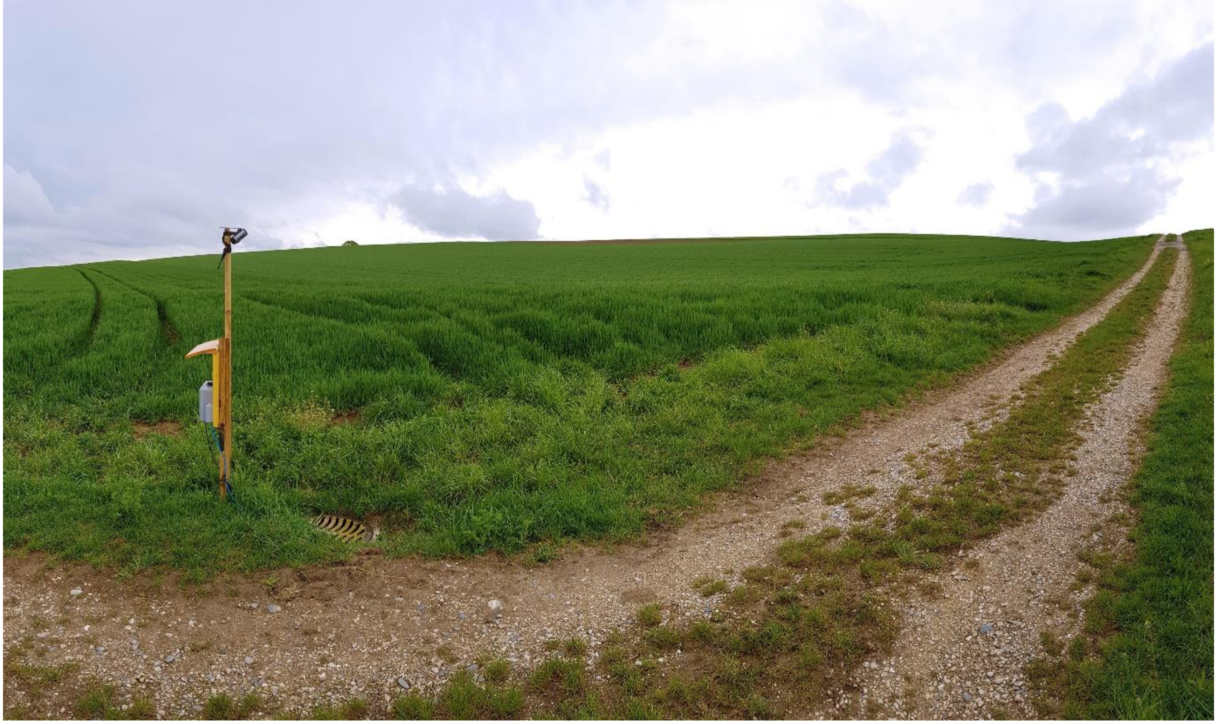

Figure A3: Sampling site I3. The site is situated between a steep einkorn wheat field and a steep farm track. The inlet is separated from the farm track by a grass buffer of approximately 0.5 m width. The field is separated from the farm track by a buffer strip of approximately 2 m width.

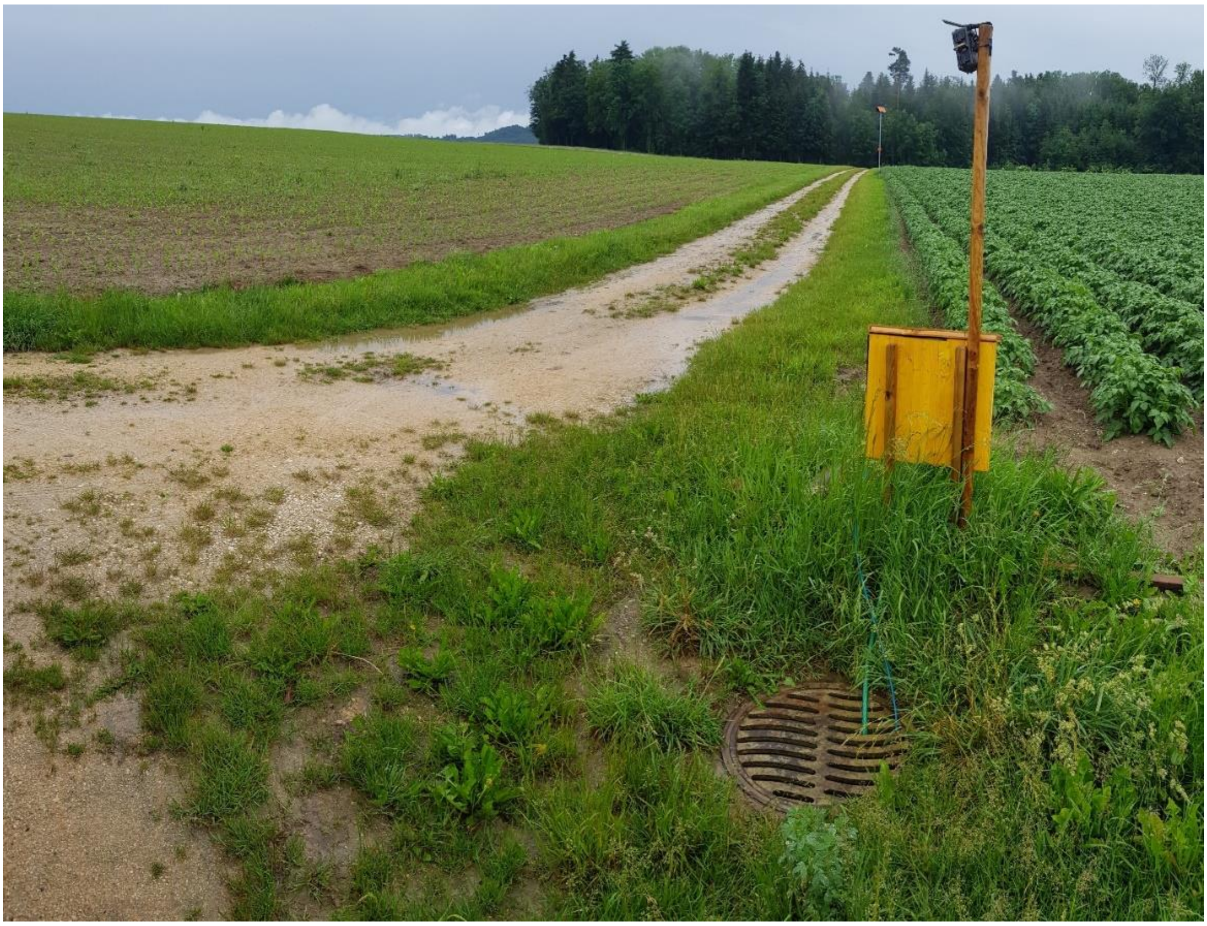

Figure A4: Sampling site I4. The site is located at a flat farm track below a steep corn field (left), and next to a flat potato field (right). The two fields are separated from the farm track by a grass buffer strip of approximately 1 m.

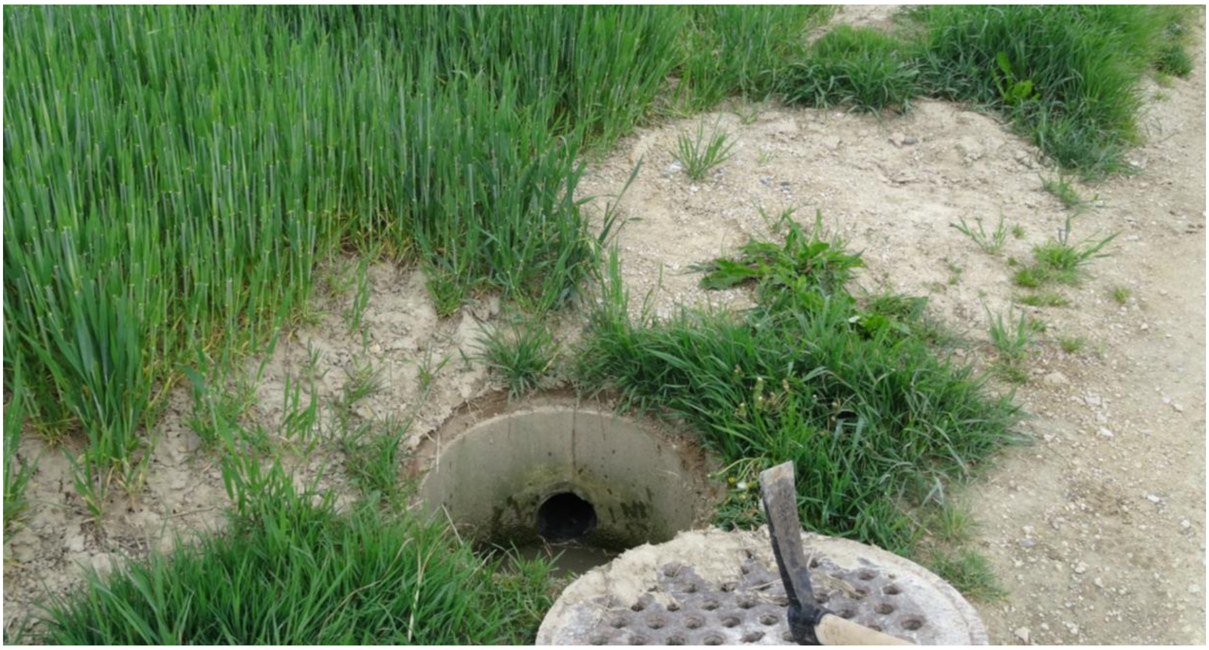

Figure A5: Picture of an inlet in the catchment. For taking the picture, the gridded lid was removed. The outlet pipe visible is the only pipe in the inlet, and drains to the stream. The water in the inlet stagnates at the height of the outlet pipe bottom.

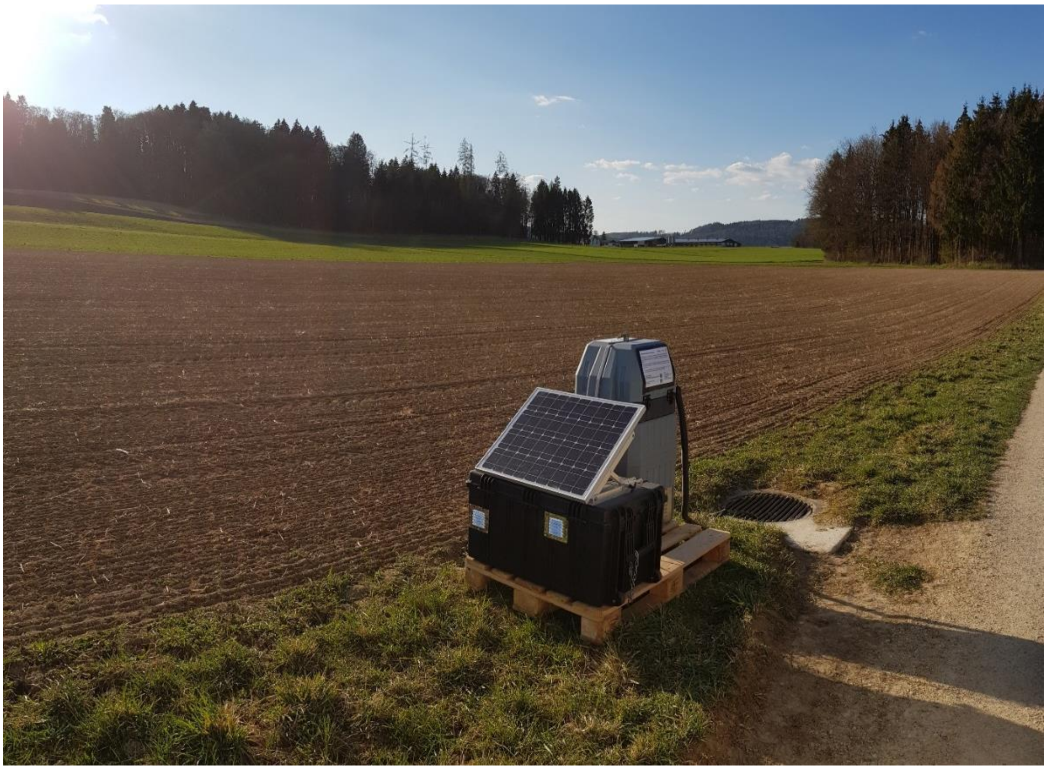

Figure A6: Outside view of sampling site CS.

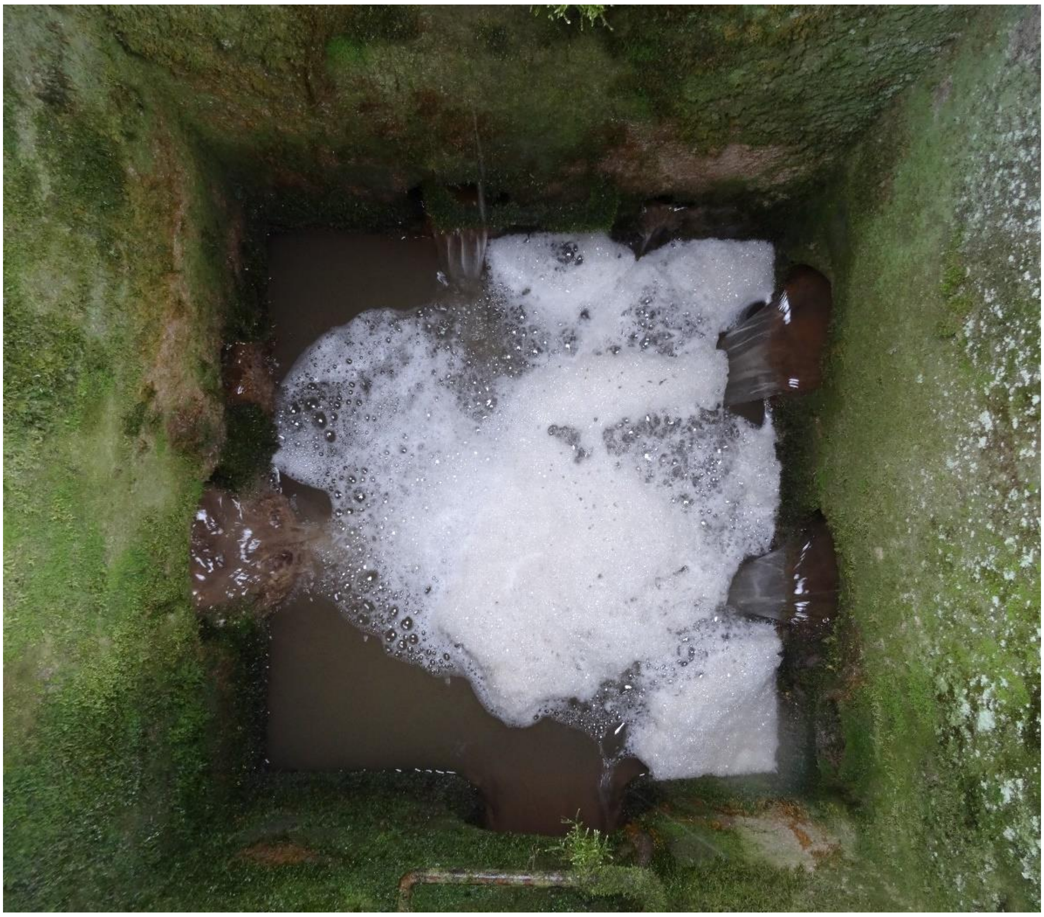

Figure A7: Inside view of sampling site CS.

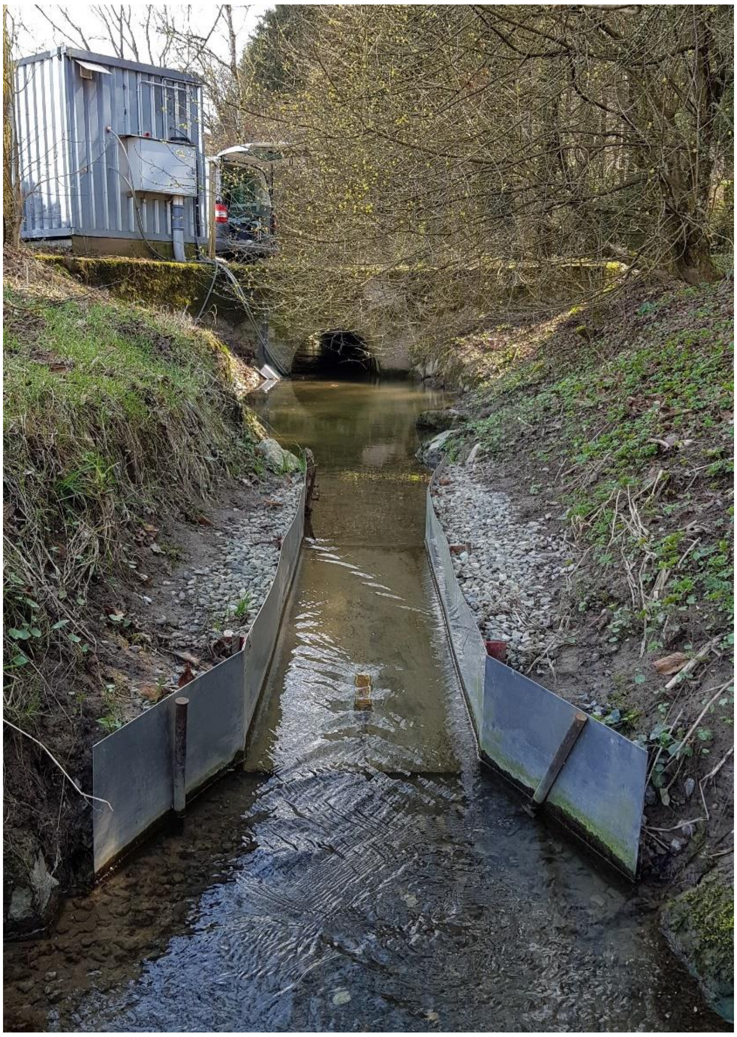

Figure A8: Sampling site ST.

A1.1.2 Water-level proportional samplers

In the following, we provide a short description of the water-level proportional samplers used in the storm drainage inlets. A detailed description of the samplers is provided in Schönenberger et al. (2020). The water-level proportional samplers consisted of a glass bottle with a volume of 1L (DURAN Weithalsglasflasche GLS 80), sealed with a screw cap (DURAN GLS80) which had two openings (Figure A9). One of the openings was equipped with a bent metal tube, the other one with a plastic tubing of 2m length (FESTO PUN 6x1-BL) connected to a needle valve (Bronkhorst precision valve, NV-004-HR).

During rain events, surface runoff entering the inlets produces a rise of the water level in the inlets. When the water level was high enough such that the samplers are submerged (this was the case at a water level of 2 cm for inlets with little runoff, and 3 cm for inlets with larger runoff), water starts to flow into the glass bottle (A) through the metal tube (C). In the bottle, the air is compressed and pressed

out of the bottle through the needle valve (E). Consequently, an equilibrium between the inflowing water volume, the outflowing air volume, and the compression of air and water in the bottle is established. As soon as this equilibrium is established, an increase of water level pressure leads to an increase in the sampling rate, and consequently, the sampling rate is proportional to the water level. The sampling stops either when the water level drops below the water inlet, or when the sampling bottle is full.

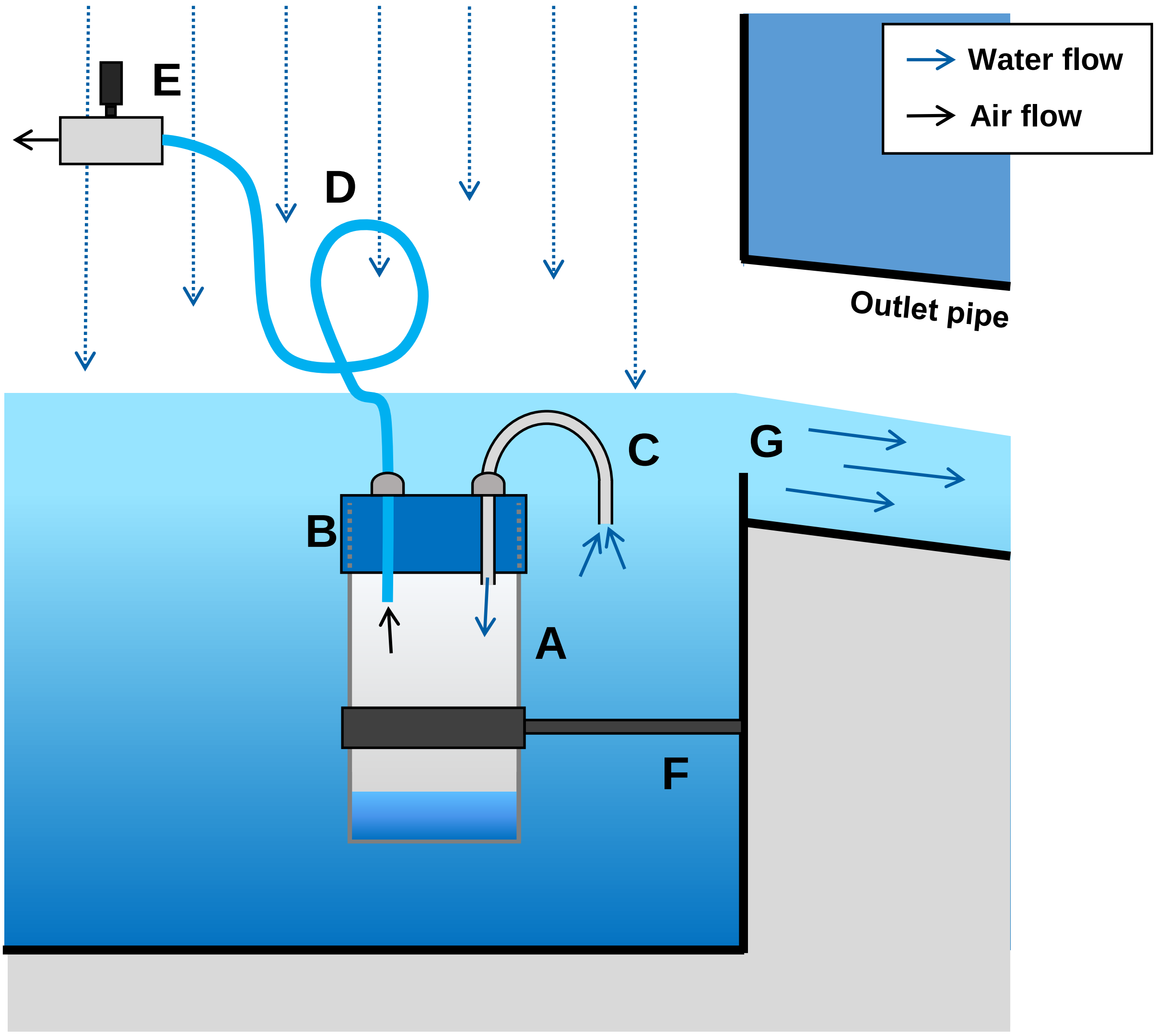

Figure A9: Water-level proportional sampler in a stormwater drainage inlet during a rain event. A: Glass bottle, B: Screw cap, C: Metal tube, D: Plastic tubing, E: Needle valve. F: Fixation of the sampler. G: Weir. Adapted from Schönenberger et al. (2020).

A1.1.3 Sampling strategy

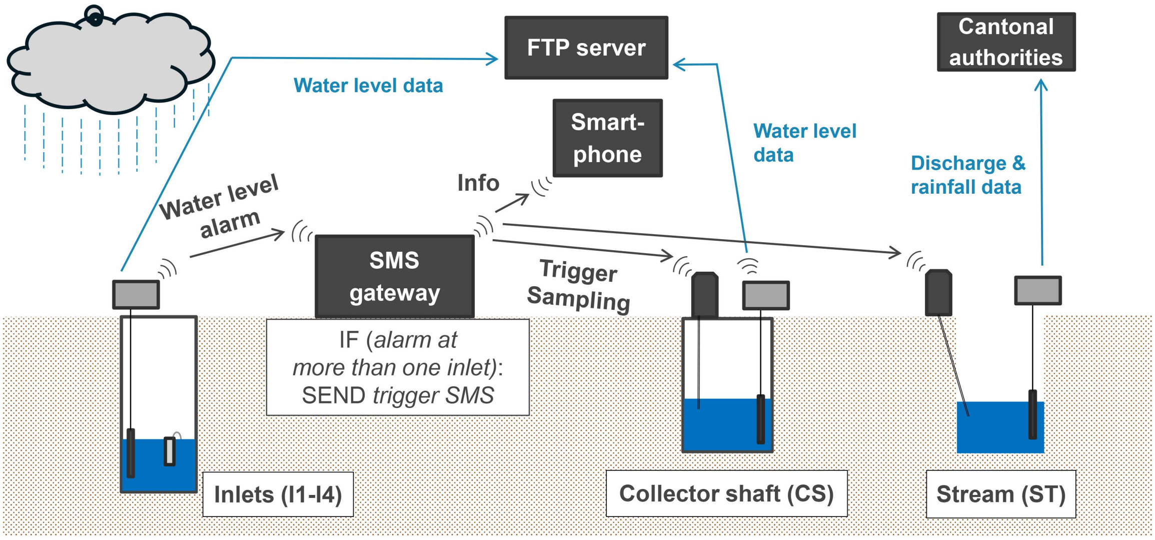

Figure A10: Illustration of the event-based sampling strategy. When the water level threshold was exceeded in at least two of the inlets (I1-I4), the automatic samplers at the collector shaft (CS) and the stream (ST) were triggered via the GPRS module to start sampling. Additionally, water level data and the information about the triggering of the samplers were sent to the research institute via the GPRS modules.

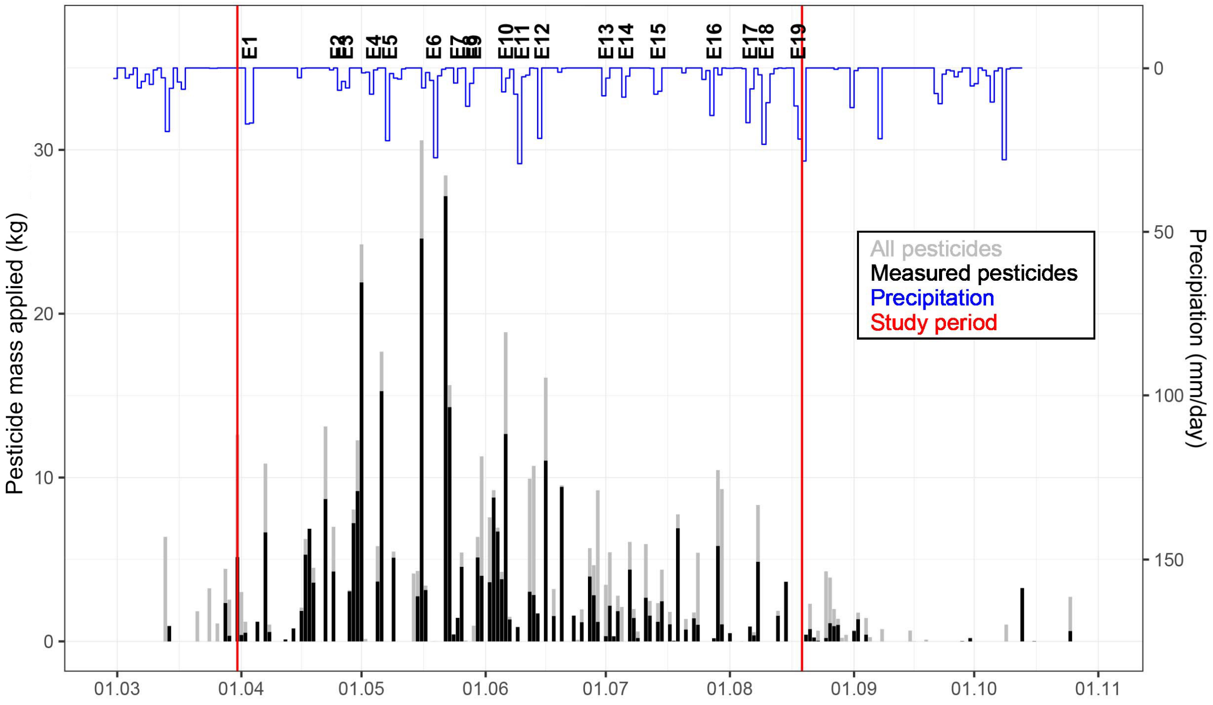

Figure A11: Total masses of pesticides applied in the study catchment per day in 2019 (kg). The red lines depict the start and the end of the study period (01.04.2019 and 20.08.2019). Grey bars show the total pesticide mass applied on the respective day. Black bars show the total pesticide mass applied for only those substances that were analysed within this study. Oils used as pesticides (e.g. paraffin oil, rapeseed oil) were excluded from the analysis. E1 to E19 indicate the rain events sampled in this study.

A1.1.4 Field mapping

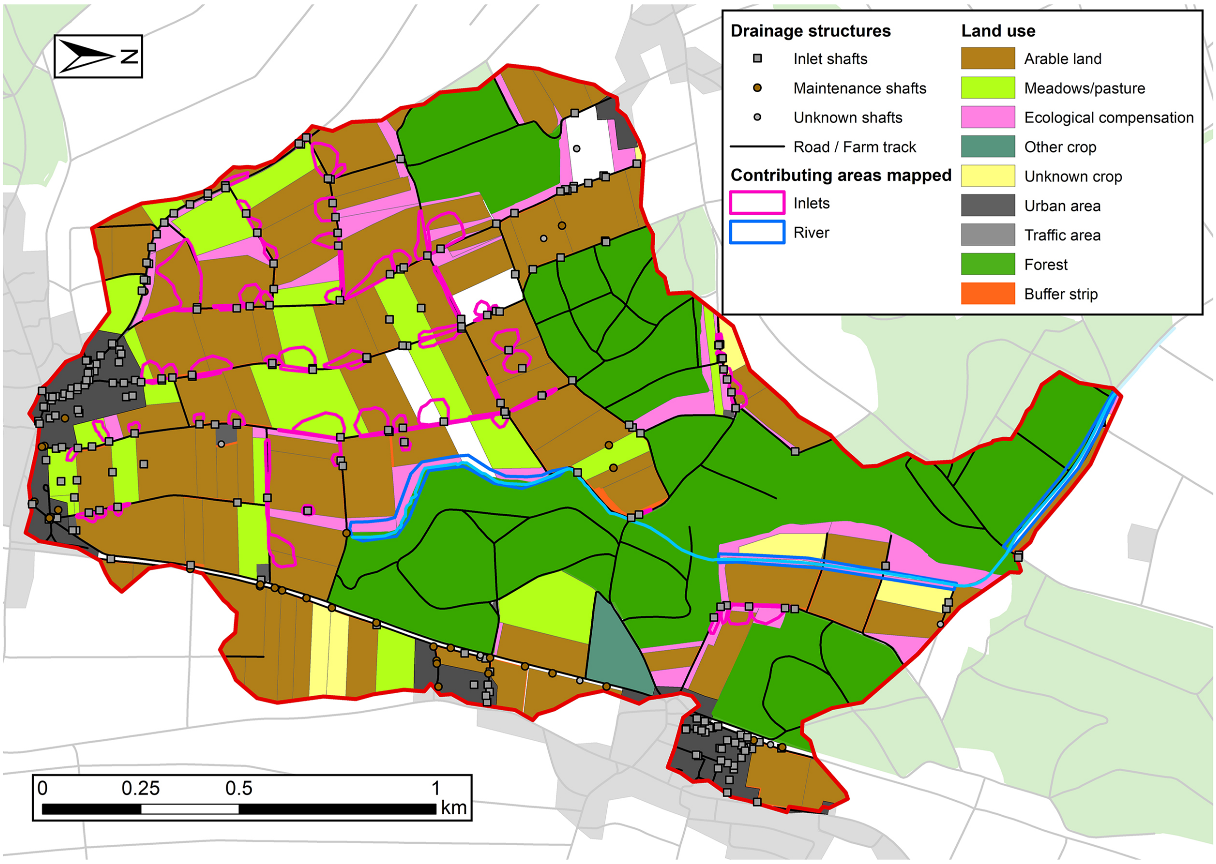

Figure A 12: Map of surface runoff flow paths during a snowmelt event on 12 March 2018. The mapped contributing areas are minimal contributing areas and may be much larger in reality. They were only mapped for some of the inlets and may differ for other events. Source of background map: Swisstopo (2020).

A1.2 Chemical analysis

In the following, further details on the chemical analysis procedure are given. A table with all substances measured is given below (Table A3).

Field samples were stored at -20° until further process. After thawing, the sample was shaken, and 1.5 ml sample was transferred to a 1.5 ml vial and closed with a magnetic starburst cap. The sample preparation was achieved through a fully automated workflow using a PAL RTC (CTC analytics AG, Switzerland) equipped with a dilutor tool, centrifuge, C-stack, fast wash station and an injection valve. After centrifugation of the samples (5 min at 2000g), 600 µL of supernatant was aspirated by the dilutor. The dilutor needle was washed by aspirating 10 µL of nanopure water at the fast wash station. Afterwards, 10 µL of a standard mix containing 84 isotopic labelled internal standards (ISTD, details Table A3) at a concentration of 0.01 mg/L was added to the dilutor tubing and separated again with 10 µL of nanopure water. Depending on the sample type either an exact volume of standard solution (concentration: 0.06, 0.006 and 0.0006 mg/L) was added and then equalized by an ethanol volume ranging from 0 to 50 µL

(standards and spiked samples) or just the equalization volume of 50 µL Ethanol was added (samples and blanks). This ensured constant sample constitution and an organic content of ~5%. The entire sample volume of 670 µL was then transferred into an empty vial equipped with a slitted septa and mixed by aspirating and dispensing the dilutor. Sample preparation occurred interlaced with sample acquisition. During one measurement four samples were prepared as described above.

For the measurement, a volume of 100 µL was injected on to the chromatographic system. Chromatographic separation was achieved using a reversed phase column (Atlantis T3, particle size 3µm, 3.0×150 mm, Waters) and a linear water-methanol gradient, both acidified with 0.1% formic acid. The flow rate was 0.3 µL/min and the column temperature was 30°C. The gradient was as follows: 0-1.5 min constant at 0% methanol, 1.5-18.5 min linear gradient to 95% methanol, 18.5-30.5 min constant at 95% methanol followed by equilibration (0% methanol) for 3.5 min. Data acquisition was accomplished with a Lumos Fusion (Thermo Fisher Scientific) running in positive and negative electrospray ionization mode separately (spray voltage: 3500 V in pos, 3000 V in neg). Full scans were recorded with a resolution of 240’000 (at m/z 200) and mass range 100-1000 m/z followed by three data-dependent MS2 scans using higher energy collision-induced dissociation (HCD) at a resolution of 15’000 (at m/z 200).

Peak integration was performed using TraceFinder 5.1 with a mass tolerance of 5 ppm. Substance confirmation occurred through comparison of the retention time, exact mass and fragment spectra with reference material. Quantification was achieved with a linear calibration curve using the peak area ratio of the analyte and ISTD. The calibration curve ranged from 10 to 5000 ng/L. For compounds without structurally identical ISTD, a closely eluting or structurally similar ISTD was chosen to reach the best relative recovery (close to 100% in spiked samples). The assignment of ISTDs and relative recoveries are shown in Table A3. For those compounds, the concentrations were corrected by the relative recovery. The LOQ was determined by the concentration of the lowest standard that was still detected with a good chromatographic peak (at least 4 sticks) and whose area was at least 4 times higher than in laboratory or field blank samples. The lowest calibrations standard value was then corrected by the matrix factor for the final LOQ. For 80% of the compounds, the LOQ was 20 ng/L or lower (see Table A3). For quality control, 54 laboratory and 11 field blanks were measured and taken into account for the LOQ. Additionally, 18 random samples were spiked with 50 and 500 ng/L to determine the relative recovery and matrix suppression.

A1.3 Data analysis

A1.3.1 Surface runoff connectivity model

Table A1: Parameters of the surface runoff connectivity model used for determining catchments of inlets, river, and internal sinks.

Parameter	Value
Hedge infiltration	No hedges in the catchment
Forest infiltration width	No infiltration in forests
Road carving depth	25 cm
Sink depth	25 cm
Shortcut definition	Only inlets act as shortcut
Maximal flow distance	No restriction on maximal flow distance

A1.3.2 Discharge measurement in inlets

As mentioned in Sect. 2.4.4, the discharge in the inlets was determined using water level measurements combined with triangular weirs for which a rating curve was calibrated. The weirs consisted of stainless chromium steel plates with two triangles of different slopes cut out (see Figure A13), and were installed in front of the outlet pipes of the measured inlets. The space between the outlet pipe and the weir was sealed with rubber.

For determining the rating curve of the weirs, their wetted area was split into three areas A, B, and C, as shown in Figure A13. For each area, a separate rating curve was determined and the rating curve of the weir was calculated by summing up the contributions of all three areas (eq. A2.1 to A2.3).

Area A was defined as the wetted area for water levels (p) smaller or equal to the water level at the slope changing point of the triangular weir (p_{wc}). For this area, the weir corresponds to a normal triangular weir and its rating curve can be described according to eq. A1.1 (Aigner 2008). Area B was defined as the wetted area between the slope changing point of the triangular weir (p_{wc}) and the water level up to which the discharge was calibrated ($p_{cal,max}$). We neglected the influence of flow in area A on the flow in area B and assumed that the shape of the rating curve of area B corresponded to the curve of a trapezoid weir (eq. A1.2, Aigner 2008).

For area C (water levels higher than $p_{cal,max}$), we created three different assumptions, corresponding to a minimum (Q_{min}), moderate (Q_{mod}) and high (Q_{high}) discharge estimate (example, see Figure A14). For the minimum estimate (eq. A2.1), we set the upper discharge limit to the maximal discharge for which the weir was calibrated $Q(p_{cal,max})$. For the moderate estimate (eq. A2.2), we assumed the shape of the rating curve in area C to correspond to the curve of a circular weir (eq. A1.3, Aigner 2008). The upper discharge limit was set to the discharge calculated for the water level at the upper weir end (p_{max}). For

the high estimate (eq. A2.3), we extrapolated the shape of the rating curve of area B (eq. A1.2) and set an upper discharge limit to the discharge calculated for p_{max}.

The weir discharge coefficients μ_{tri} and μ_{tra} were calibrated by pouring known discharges into an inlet with a tube and measuring the emerging water levels. Since no discharges corresponding to water levels higher than $p_{cal,max}$ could be produced with the tube, the coefficient μ_{cir} was calibrated using only one data point (i.e. the data point at water level $p_{cal,max}$).

$$Q_{tri}(p) = \frac{8}{15}\mu_{tri} \cdot \sqrt{2g} \cdot \frac{b}{2 \cdot p_{wc}} \cdot p^{\frac{5}{2}} \tag{A1.1}$$

$$Q_{tra}(p) = \frac{2}{3}\mu_{tra} \cdot \sqrt{2g} \cdot b \cdot (p - p_{wc})^{\frac{1}{5}} \cdot \left(1 + \frac{4}{5}\frac{(p-p_{wc}) \cdot w}{b \cdot h}\right) \tag{A1.2}$$

$$Q_{cir}(p) = \mu_{cir} \cdot \sqrt{2g} \cdot d^{\frac{2}{3}} \cdot \left(\left(p - p_{cal,max} + r\right)^{\frac{11}{6}} - r^{\frac{11}{6}}\right) \tag{A1.3}$$

$$Q_{min} = \begin{cases} Q_{tri}(p) & | \; p \leq p_{wc} \\ Q_{tri}(p_{wc}) + Q_{tra}(p) & | \; p_{wc} < p \leq p_{cal.max} \\ Q_{tri}(p_{wc}) + Q_{tra}(p_{cal.max}) & | \; p > p_{cal.max} \end{cases} \tag{A2.1}$$

$$Q_{mod} = \begin{cases} Q_{tri}(p) & | \; p \leq p_{wc} \\ Q_{tri}(p_{wc}) + Q_{tra}(p) & | \; p_{wc} < p \leq p_{cal.max} \\ Q_{tri}(p_{wc}) + Q_{tra}(p_{cal.max}) + Q_{cir}(\min(p, p_{max})) & | \; p > p_{cal.max} \end{cases} \tag{A2.2}$$

$$Q_{high} = \begin{cases} Q_{tri}(p) & | \; p \leq p_{wc} \\ Q_{tri}(p_{wc}) + Q_{tra}(p) & | \; p_{wc} < p \leq p_{cal.max} \\ Q_{tri}(p_{wc}) + Q_{tra}(\min(p, p_{max})) & | \; p > p_{cal.max} \end{cases} \tag{A2.3}$$

with:

Q_{tri}, Q_{tra}, Q_{cir}:	Rating curves for the triangular, trapezoid, and circular part of the weir ($m^3 \, s^{-1}$)
Q_{min}, Q_{mod}, Q_{high}:	Minimal, moderate, and high discharge estimate ($m^3 \, s^{-1}$)
p:	Water level (m)
p_{wc}:	Water level at the slope changing point of the triangular weir (= 0.03 m)
$p_{cal,max}$:	Maximal water level up to which the weir was calibrated (m)
p_{max}:	Water level at the upper end of the weir (= 0.075 m)
b, h, w:	Dimensions of the triangular weir (m) (see Figure A13)
d:	Diameter of the outlet pipe (m)
r:	Radius of the outlet pipe (m)
g:	Acceleration due to gravity (= 9.807 $m \, s^{-2}$)
μ_{tri}, μ_{tra}, μ_{cir}:	Weir discharge coefficients (-)

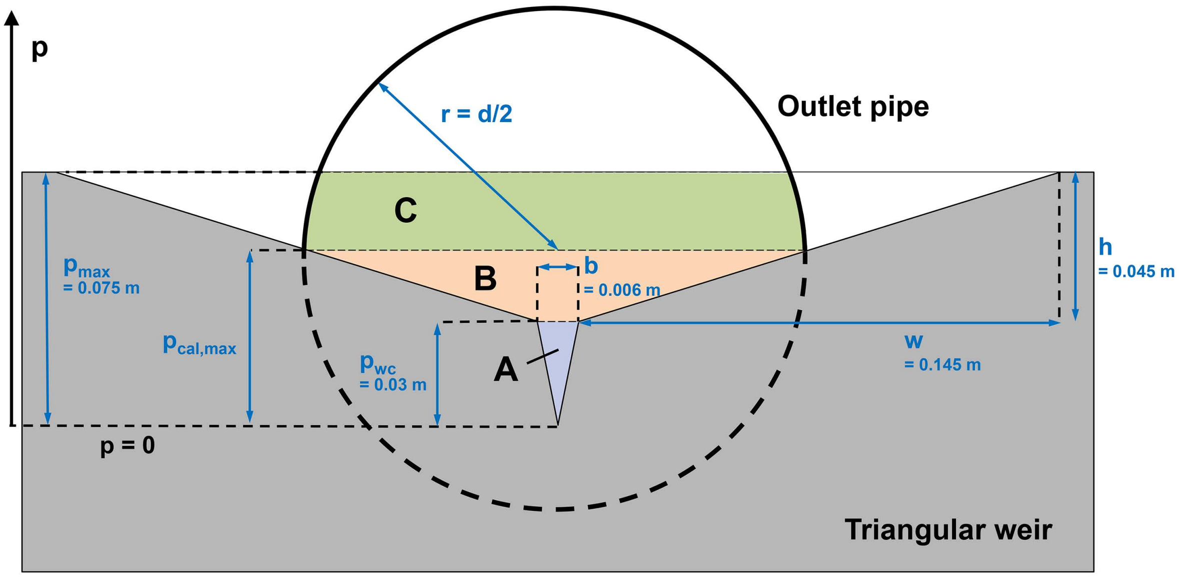

Figure A13: Dimensions of the triangular weir (grey area) and the subareas used for rating curve determination.

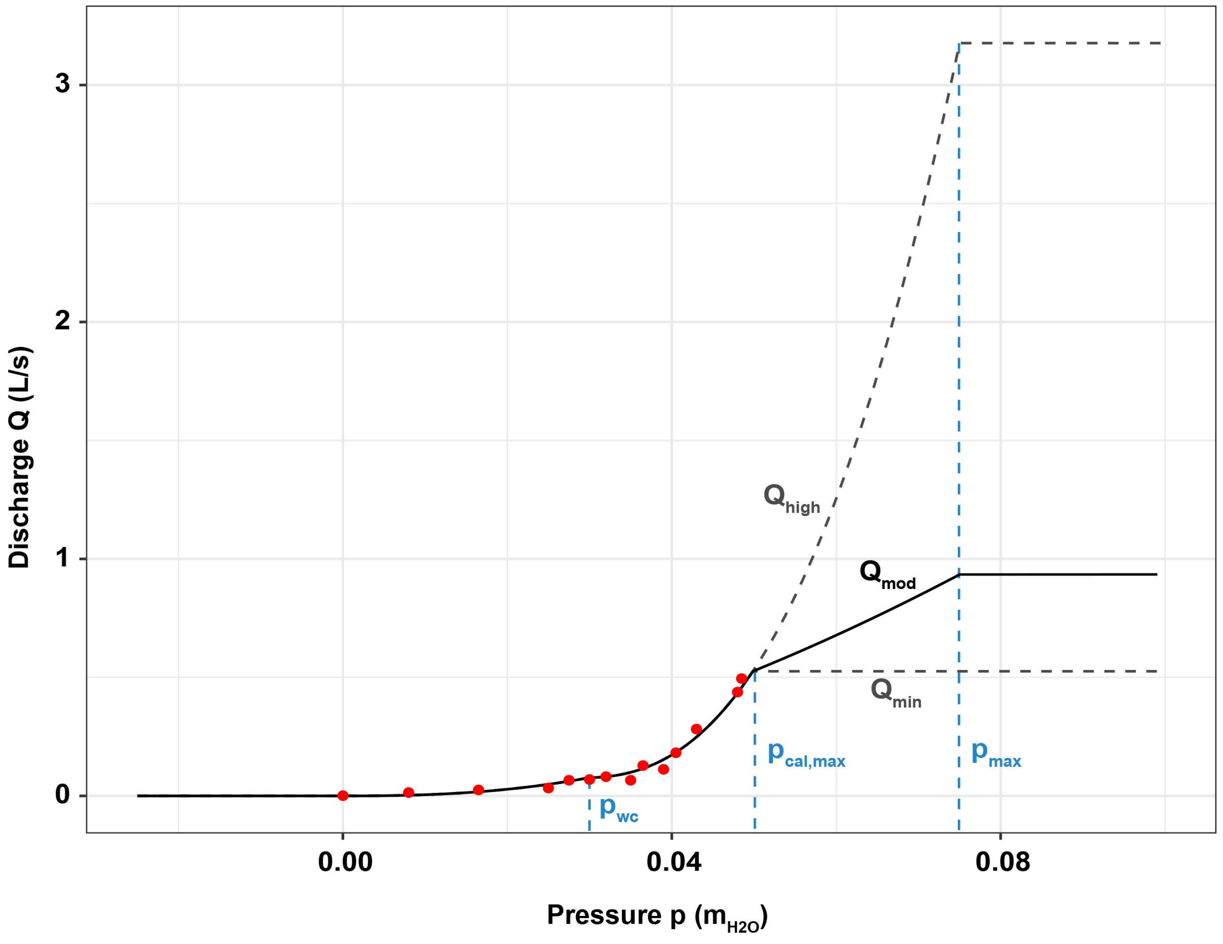

Figure A14: Rating curve of the triangular weir installed in inlet 3. The black solid line represents the moderate discharge estimate (Q_{mod}) and the black dashed lines represent the minimal and high discharge estimates (Q_{min} and Q_{high}). The red dots show the measurements used for calibration of the rating curve.

To compare the discharge in the inlets and the stream, we calculated the ratio between the discharge sum of all four inlets to the discharge in the stream (r_Q) (eq. A3.1). For the discharge measured in the stream Q_{stream}, the cantonal authorities provided no information on uncertainty. Expecting that the relative uncertainty of the discharge through inlets is much larger than the uncertainty in stream discharge, we neglected the latter.

$$\mathbf{r_Q} = \begin{pmatrix} r_{Q,min} \\ r_{Q,mod} \\ r_{Q,high} \end{pmatrix} = \frac{\sum_{i=1}^{4} \mathbf{Q_{inl,i}}}{Q_{stream}} = \frac{\sum_{i=1}^{4} \begin{pmatrix} Q_{inl,i,min} \\ Q_{inl,i,mod} \\ Q_{inl,i,high} \end{pmatrix}}{Q_{stream}} \quad \text{(A3.1)}$$

$\mathbf{Q_{inl,i}}$: Discharge estimates (minimum, moderate, high) in inlet i (m^3 s^{-1})

Q_{stream}: Discharge in the stream (m^3 s^{-1})

Additionally, we calculated the ratio between the discharge sum in inlets and the fast discharge in the stream ($r_{Q,fast}$) (eq. A3.2).

$$\mathbf{r_{Q,fast}} = \begin{pmatrix} r_{Q,fast,min} \\ r_{Q,fast,mod} \\ r_{Q,fast,high} \end{pmatrix} = \frac{\sum_{i=1}^{4} \mathbf{Q_{inl,i}}}{\mathbf{Q_{stream,fast}}} = \frac{\sum_{i=1}^{4} \begin{pmatrix} Q_{inl,i,min} \\ Q_{inl,i,mod} \\ Q_{inl,i,high} \end{pmatrix}}{\begin{pmatrix} Q_{stream,fast,high} \\ Q_{stream,fast,mod} \\ Q_{stream,fast,low} \end{pmatrix}} \quad \text{(A3.2)}$$

$\mathbf{Q_{stream,fast}}$: Fast discharge fraction estimates in the stream (m^3 s^{-1})

We estimated the fast discharge fraction in the stream using a recursive filter technique (Lyne and Hollick, 1979) for discharge separation (function “BaseflowSeparation” of the R package “EcoHydRology”, version 0.4.12.1, Fuka et al. (2018)). We used three different filter parameters (0.9, 0.925, and 0.95; see Nathan and Mcmahon (1990)) to come up with a low, moderate, and high estimate of the fast discharge fraction.

Based on the discharge measurements in the four inlets, we estimated the total discharge flowing through all inlets in the catchment $Q_{inl,tot}$. For this, we used three simple extrapolation methods. In the first two methods, we assumed that the discharge in an inlet is proportional to the road area (eq. A4.1) or to the agricultural area connected to the inlet (eq. A4.2). In the third method, we assumed that the discharge is proportional to the number of inlets (eq. A4.3). These three methods are meant to provide a rough estimate of the total discharge and various parameters influencing the total discharge (such as slope, soil permeability, crop types, spatial distribution of rainfall) were not taken into account here.

$$\mathbf{Q_{inl,tot,road}} = \frac{\sum_{i=1}^{4} A_{road,i}}{A_{road,tot}} \cdot \sum_{i=1}^{4} \mathbf{Q_{inl,i}} \quad \text{(A4.1)}$$

$A_{road,i}$: Road area connected to inlet i (m^2)
$A_{road,tot}$: Total road area connected to inlets in the catchment (m^2)
$Q_{inl,i}$: Discharge in inlet i (m^3 s^{-1})

$$\mathbf{Q_{inl,tot,agri}} = \frac{\sum_{i=1}^{4} A_{agri,i}}{A_{agri,tot}} \cdot \sum_{i=1}^{4} \mathbf{Q_{inl,i}} \quad \text{(A4.2)}$$

$A_{agri,i}$: Agricultural area connected to inlet i (m^2)
$A_{agri,tot}$: Total agricultural area connected to inlets in the catchment (m^2)

$$\mathbf{Q_{inl,tot,num}} = \frac{n_{inl,tot}}{n_{inl,measured}} \cdot \sum_{i=1}^{4} \mathbf{Q_{inl,i}} = \frac{158}{4} \cdot \sum_{i=1}^{4} \mathbf{Q_{inl,i}} \quad \text{(A4.3)}$$

$n_{inl,\ measured}$: Number of inlets with discharge measurements (-)
$n_{inl,\ tot}$: Total number of inlets in the catchment (-)

A1.3.3 Model of concentrations in inlets

Table A2: Overview over the variables used for building the linear mixed model.

Variable	Abbre-viation	Type	Discrete/ Continuous	Unit	Range/ Categories
Inlet concentration	$\log_{10}(c)$	Response variable	Continuous	ng L^{-1}	$\log_{10}$([5, 62000])
Time since application	t_{appl}	Fixed effect	Continuous	days	[1.2, 142]
Amount of pesticide applied per area	$\log_{10}(m_{appl})$	Fixed effect	Discrete	g ha^{-1}	$\log_{10}$ ([1.2, 1600])
Freundlich adsorption coefficient normalized to organic carbon content	$\log_{10}(K_{foc})$	Fixed effect	Discrete	mg L^{-1}	$\log_{10}$ ([20, 4900])
Octanol-water partition coefficient	$\log_{10}(K_{ow})$	Fixed effect	Discrete	-	[-1.2, 4.7]
Half-life in water	$DT_{50,\ water}$	Fixed effect	Discrete	days	[0.30, 92]
Half-life in soil	$DT_{50,\ soil}$	Fixed effect	Discrete	days	[0.34, 500]
Moderate estimate of the discharge in the inlet during the event	$\log_{10}(Q_{mod})$	Fixed effect	Continuous	L	$\log_{10}$ ([0,8500])
Potential transport processes involved	$p_{transport}$	Fixed effect	Categorial	-	(A, B, C, D) (see Sect. 2.4.3)
Inlet sampled	i	Random effect	Categorial	-	(1, 2, 3, 4)

Table A3: List of the 51 substances analysed. P: Pesticide, TP: Transformation product, LOQ: Limit of quantification, RR: Relative recovery, LMM: Substance used in the linear mixed model. The samples were measured in three sets. Below, we therefore report the LOQs and RRs for each set.

Substance measured	CAS number	InChIKey	Sub-stance type	Pesticide class	LOQ Set 1 (ng/L)	LOQ Set 2 (ng/L)	LOQ Set 3 (ng/L)	RR set 1 (%)	RR set 2 (%)	RR set 3 (%)	Semi-quanti-tative	Internal standard	LMM
Azoxystrobin	131860-33-8	WFDXOXNFNRHQEC-GHRIWEEISA-N	P	Fungicide	7	9	6	78	82	97	no	Azoxystrobin D4	
Bixafen	581809-46-3	LDLMOOXUCMHBMZ-UHFFFAOYSA-N	P	Fungicide	8	20	5	80	98	105	no	Flufenacet D5	x
Boscalid	188425-85-6	WYEMLYFITZORAB-UHFFFAOYSA-N	P	Fungicide	10	10	4	89	92	93	no	Boscalid D4	
Carfentrazone-ethyl	128639-02-1	MLKCGVHIFJBRCD-UHFFFAOYSA-N	P	Herbicide	10	20	15	50	83	74	no	Epoxiconazole D4	
Chlortoluron	15545-48-9	JXCGFZXSOMJFOA-UHFFFAOYSA-N	P	Herbicide	10	6	6	83	97	101	no	Chlortoluron D6	
Cymoxanil	57966-95-7	XERJKGMBORTKEO-VZUCSPMQSA-N	P	Fungicide	9	9	9	76	87	174	partially	Metamitron D5	x
Cyproconazole	94361-06-5	UFNOUKDBUJZYDE-UHFFFAOYSA-N	P	Fungicide	8	8	4	83	100	102	no	Dimethenamid D3	
Difenoconazole	119446-68-3	BQYJATMQXGBDHF-UHFFFAOYSA-N	P	Fungicide	25	8	8	121	106	114	no	Pyraclostrobin D3	x
Diflufenican	83164-33-4	WYEHFWKAOXOVJD-UHFFFAOYSA-N	P	Herbicide	500	200	100	81	143	103	no	Metrafenone D9	
Dimethachlor	50563-36-5	SCCDDNKJYDZXMM-UHFFFAOYSA-N	P	Herbicide	6	9	10	79	86	106	partially	Dimethenamid D3	
Dimethenamid	87674-68-8	JLYFCTQDENRSOL-UHFFFAOYSA-N	P	Herbicide	9	8	4	89	102	98	no	Dimethenamid D3	x
Epoxiconazole	133855-98-8	ZMYFCFLJBGAQRS-UHFFFAOYSA-N	P	Fungicide	7	8	6	75	78	99	no	Epoxiconazol D4	x
Ethofumesate	26225-79-6	IRCMYGHHKLLGHV-UHFFFAOYSA-N	P	Herbicide	9	20	10	88	109	87	no	Azoxystrobin D4	x
Fenpropimorph	67306-03-0	RYAUSSKQMZRMAI-ALOPSCKCSA-N	P	Fungicide	6	5	10	64	105	93	no	Metribuzin D3	x
Florasulam	145701-23-1	QZXATCCPQKOEIH-UHFFFAOYSA-N	P	Herbicide	100	50	20	73	91	95	no	2,4-D3	x
Fluazifop (free acid)	69335-91-7	YUVKUEAFAVKILW-UHFFFAOYSA-N	P	Herbicide	40	9	10	100	103	98	no	Mecoprop D6	
Fluazinam	79622-59-6	UZCGKGPEKUCDTF-UHFFFAOYSA-N	P	Fungicide	200	8	4	345	123	121	no	Fipronil 13C15N2	
Flufenacet	142459-58-3	IANUJLZYFUDJIH-UHFFFAOYSA-N	P	Herbicide	8	20	6	84	98	91	no	Flufenacet D4	
Fluopicolide	239110-15-7	GBOYJIHYACSLGN-UHFFFAOYSA-N	P	Fungicide	8	9	2	97	99	109	no	Dimethenamid D3	

Flupyrsulfuron-methyl	14470-53-4	DTVOKYWXACGVGO-UHFFFAOYSA-N	P	Herbicide	10	9	10	66	105	102	no	Boscalid D4	
Foramsulfuron	173159-57-4	PXDNXJSDGQBLKS-UHFFFAOYSA-N	P	Herbicide	9	9	4	80	93	97	partially	Metribuzin D3	
Iodosulfuron-methyl	185119-76-0	VWGAYSCWLXQJBQ-UHFFFAOYSA-N	P	Herbicide	10	15	10	47	102	100	no	Azoxystrobin D4	x
Isoproturon	34123-59-6	PUIYMUZLKQOUOZ-UHFFFAOYSA-N	P	Herbicide	2	4	6	82	94	103	partially	Isoproturon D5	
Lenacil	2164-08-1	ZTMKADLOSYKWCA-UHFFFAOYSA-N	P	Herbicide	10	15	8	88	100	100	no	Lenacil(cyclohexyl) D4	x
Mandipropamid	374726-62-2	KWLVWJPJKJMCSH-UHFFFAOYSA-N	P	Fungicide	8	9	4	92	108	117	no	Dimethenamid D3	
Mecoprop	93-65-2	WNTGYJSOUMFZEP-UHFFFAOYSA-N	P	Herbicide	100	40	5	84	109	95	no	Mecoprop D6	x
Mesosulfuron-methyl	74223-64-6	RSMUVYRMZCOLBH-UHFFFAOYSA-N	P	Herbicide	8	8	9	89	99	120	no	Dimethenamid D3	x
Metamitron	41394-05-2	VHCNQEUWZYOAEV-UHFFFAOYSA-N	P	Herbicide	20	7	8	89	105	109	no	Metamitron D5	x
Metolachlor	51218-45-2	WVQBLGZPHOPPFO-UHFFFAOYSA-N	P	Herbicide	8	8	4	91	85	95	no	Metolachlor D6	
Metrafenone	220899-03-6	AMSPWOYQQAWRRM-UHFFFAOYSA-N	P	Fungicide	15	9	6	83	95	93	no	Metrafenone D9	x
Metribuzin	21087-64-9	FOXFZRUHNHCZPX-UHFFFAOYSA-N	P	Herbicide	9	7	4	84	95	99	no	Metribuzin(S-methyl-D3)	x
Napropamide	15299-99-7	WXZVAROIGSFCFJ-UHFFFAOYSA-N	P	Herbicide	8	10	6	71	88	103	no	Terbutylazin D5	
Nicosulfuron	111991-09-4	RTCOGUMHFFWOJV-UHFFFAOYSA-N	P	Herbicide	10	8	6	92	98	92	no	Nicosulfuron D6	
Pencycuron	66063-05-6	OGYFATSSENRIKG-UHFFFAOYSA-N	P	Seed treatment	15	9	6	96	91	105	no	Metolachlor D6	x
Propamocarb	24579-73-5	WZZLDXDUQPOXNW-UHFFFAOYSA-N	P	Fungicide	5	25	50	139	135	110	no	Metribuzin(S-methyl-D3)	
Propiconazole	60207-90-1	STJLVHWMYQXCPB-UHFFFAOYSA-N	P	Fungicide	15	8	6	104	106	106	no	Metrafenone D9	x
Prosulfocarb	52888-80-9	NQLVQOSNDJXLKG-UHFFFAOYSA-N	P	Herbicide	15	8	2	104	86	88	no	Epoxiconazol D4	x
Prothioconazole	178928-70-6	MNHVNIJQQRJYDH-UHFFFAOYSA-N	P	Fungicide	100	200	100	50	74	81	no	Pyraclostrobin D3	
Pyraclostrobin	175013-18-0	HZRSNVGNWUDEFX-UHFFFAOYSA-N	P	Fungicide	50	9	15	81	101	96	no	Pyraclostrobin D3	
Spiroxamine	118134-30-8	PUYXTUJWRLOUCW-UHFFFAOYSA-N	P	Fungicide	100	25	20	153	144	61	no	Nicosulfuron D6	
Tembotrione	335104-84-2	IUQAXCIUEPFPSF-UHFFFAOYSA-N	P	Herbicide	50	8	8	62	111	109	no	Azoxystrobin D4	
Terbuthylazine	5915-41-3	FZXISNSWEXTPMF-UHFFFAOYSA-N	P	Herbicide	9	7	6	85	100	101	no	Terbutylazin D5	x

Thiacloprid	111988-49-9	HOKKPVIRMVDYPB-UHFFFAOYSA-N	P	Insecticide	7	5	15	86	114	114	partially	Clothianidin D5	
Thiamethoxam	153719-23-4	NWWZPOKUUAIXIW-DHZHZOJOSA-N	P	Insecticide	10	8	10	86	104	99	partially	Thiamethoxam D3	
Trifloxystrobin	141517-21-7	ONCZDRURRATYFI-TVJDWZFNSA-N	P	Fungicide	50	10	10	43	90	99	no	Metrafenone D9	x
CT-TP-R417888		JNMMKKYUIIQPDG-UHFFFAOYSA-N	TP	Fungicide TP	45	55	40	76	85	101	no	2,4-D3	
CT-TP-R471811		NLCNUAPJCIAONV-UHFFFAOYSA-N	TP	Fungicide TP	100	15	100	79	67	100	no	2,4-D3	
CT-TP-R611968		IODGSFOOWTXKAE-UHFFFAOYSA-N	TP	Fungicide TP	15	10	5	105	99	98	no	2,4-D3	
CT-TP-SYN507900	115044-73-0	WUYYRWIYXBUPBS-UHFFFAOYSA-N	TP	Fungicide TP	50	20	10	68	93	94	no	Fipronil 13C15N2	
Metamitron-desamino	36993-94-9	OUSYWCQYMPDAEO-UHFFFAOYSA-N	TP	Herbicide TP	10	10	3	114	113	109	no	Metamitron D5	
Metolachlor-OXA	152019-73-3	LNOOSYCKMKZOJB-UHFFFAOYSA-N	TP	Herbicide TP	10	10	10	106	103	104	no	Metolachlor D6	

A2. Results

A2.1 Hydrological behaviour of inlets

Table A4: Event rainfall needed at different inlets for surface runoff to enter the inlet. The duration of the corresponding rain events equalled 1 to 41 hours (median: 9 hours).

**Location**	**I1**	**I2**	**I3**	**I4**
Minimal amount of rainfall leading to discharge (mm)	1.3	1.5	3.6	1.3
Minimal rainfall always leading to discharge (mm)	3.5	11.5	18.8	3.5

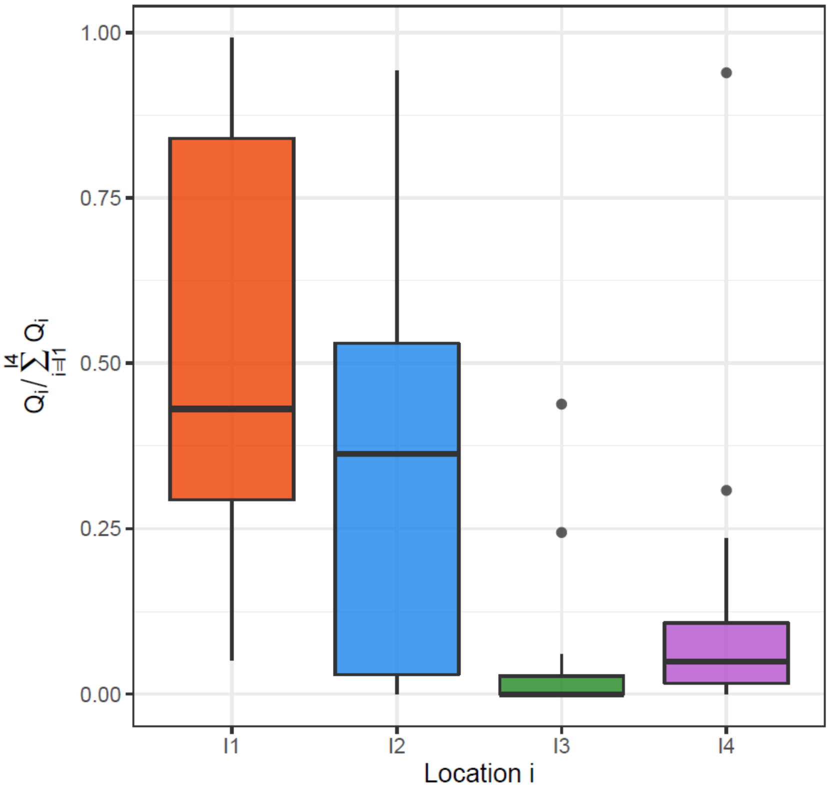

Figure A15: Distribution of the total event discharge ratio between each single inlet and the sum of all four inlets.

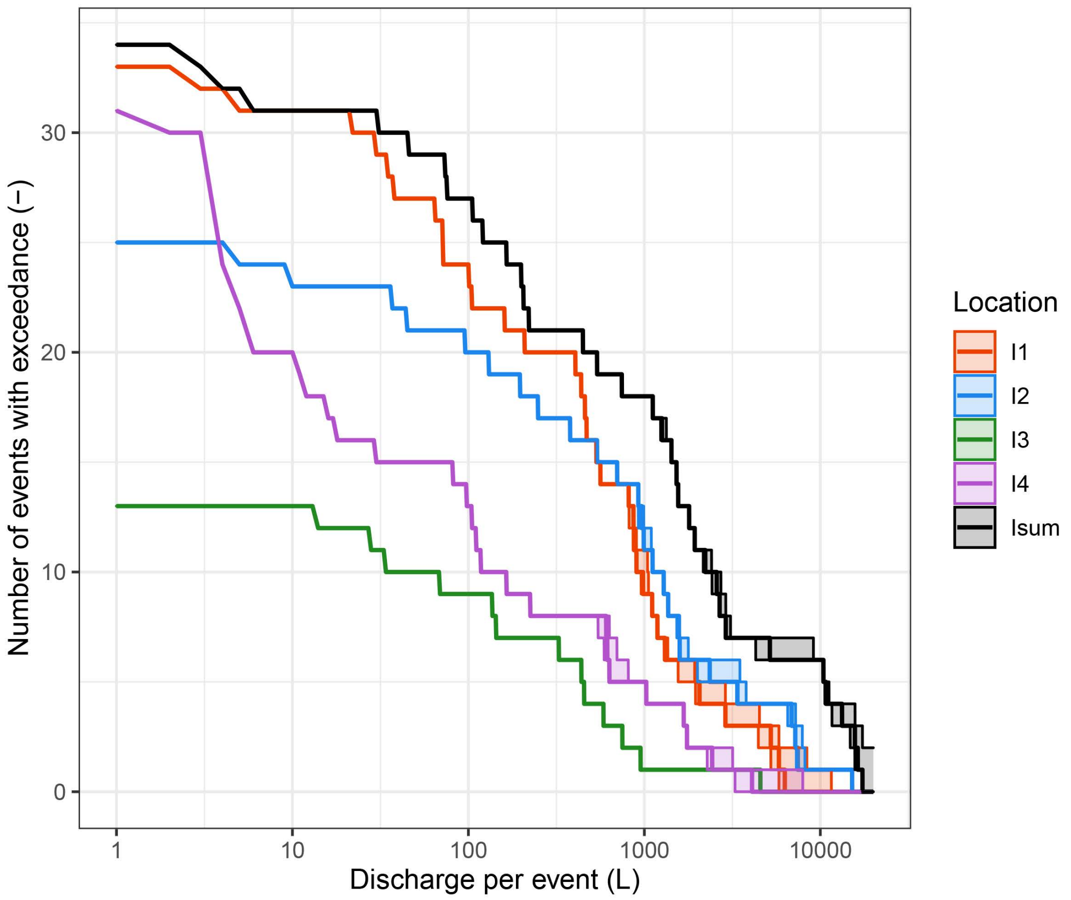

Figure A16: Number of events exceeding a given event discharge in inlets. Isum corresponds to the discharge sum of all four inlets. Bold lines indicate the moderate discharge estimate $Q_{i,e,mod}$. Thin lines indicate the minimum $Q_{i,e,min}$ and high discharge estimates $Q_{i,e,high}$.

Table A5: Fractions of fast and total discharge in the stream originating from inlets for events with total rainfall > 10 mm. Numbers report the moderate estimates. In brackets, the minimum and high estimates are given. The first column shows the measured discharge fractions in the four studied inlets, the second to fourth column show the extrapolation to all inlets in the catchment according to three different methods (i.e. proportional to the road area, the agricultural area, and the number of inlets; see eq. A4.1 to A4.3).

	Measured inlets (I1-I4)	Extrapolation to all inlets		
		Road area	Agri. area	Number of inlets
Fraction of fast discharge $r_{Q,fast}$	0.83%<br>[0.64%; 1.1%]	29%<br>[22%; 38%]	14%<br>[11%; 19%]	33%<br>[25%; 43%]
Fraction of total discharge r_Q	0.22%<br>[0.21%; 0.25%]	7.5%<br>[7.2%; 8.8%]	3.7%<br>[3.6%; 4.4%]	8.5%<br>[8.2%; 10%]

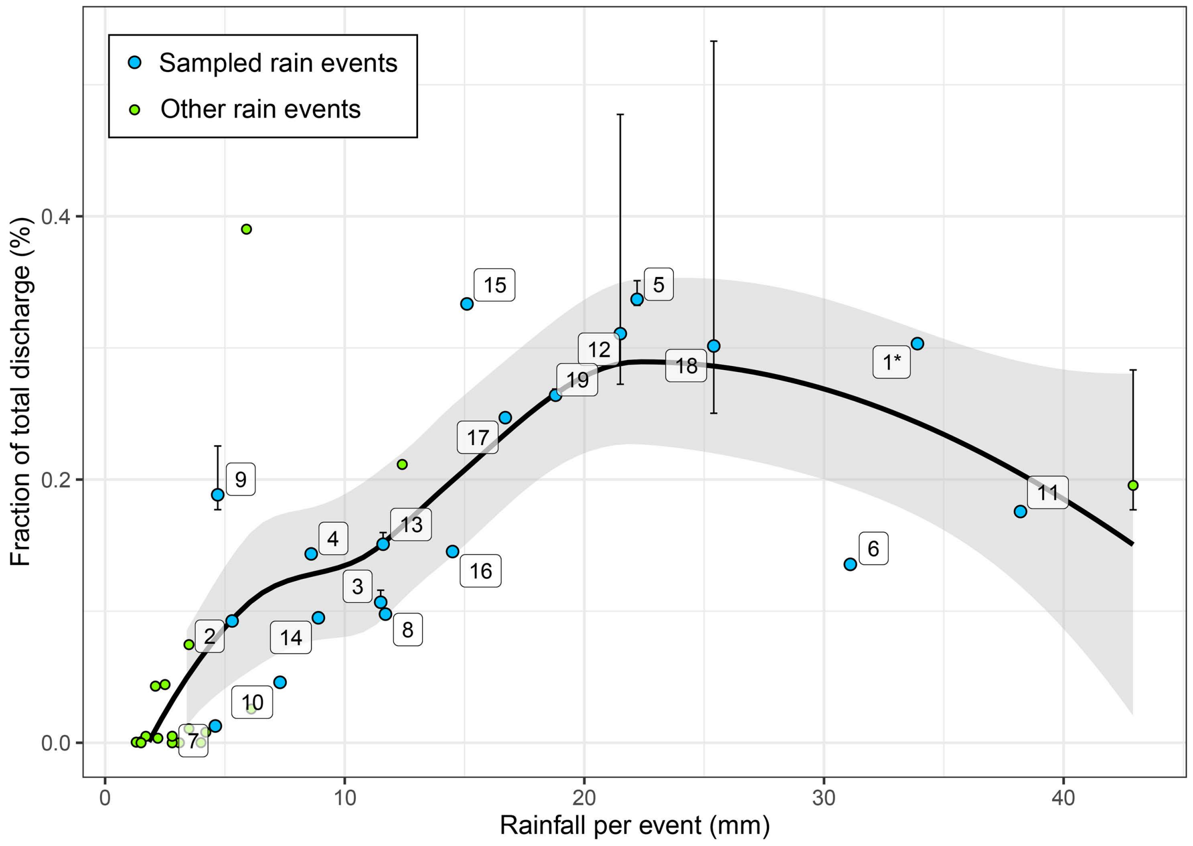

Figure A17: Ratio between total discharge originating from the four inlets and the discharge in the stream r_Q. Points indicate the moderate estimate ($r_{Q,mod}$) and the error bars correspond to the minimum and high estimate ($r_{Q,min}$ and $r_{Q,high}$). Sampling event numbers are indicated with white boxes. The black line represents a smoothed conditional mean (LOESS) of the average r_Q estimates, obtained by a locally weighted scatterplot smoothing (LOESS) using the R package ggplot2 (version 3.3.3, function geom_smooth). The grey area represents the corresponding 95% confidence interval.

A2.2 Concentrations and loads

Table A6: Overview over events analysed. I1-I4: Inlets 1 to 4, CS: collector shaft, ST: stream. The column "top ten events" shows the top ten events with respect to sum concentrations in the inlets. The letter A indicates that for the respective event samples in the collector shaft and in the stream were analysed. The letter N indicates that for the respective event only samples in the inlets were analysed. In the column "samples analysed" the sample types are indicated. c: water-level proportional composite sample, g: grab sample after the event, t: time proportional sample, b: time proportional backup sample of cantonal authorities, -: no sample available. Sampling interval: The first number indicates the sampling interval of time proportional samples in the collector shaft that were then pooled together into one composite sample with a total sampling time as indicated in brackets.

**Event**		**Rainfall**			**Discharge sum (L)**					**Sum concentrations in I1 to I4 (ng/L)**	**Top ten events**	**Samples analysed**						**Sampling interval CS (min)**
**ID**	**Starting time**	**Sum (mm)**	**Mean ints. (mm/hr)**	**Max. ints. (mm/hr)**	**I1**	**I2**	**I3**	**I4**	**ST**			**I1**	**I2**	**I3**	**I4**	**CS**	**ST**	
1	03.04 13:57	33.9	0.9	6.0	832	8519	162	122	1.4e6	1.7e4		c	c	c	c			
2	26.04 01:57	5.3	0.8	3.6	38	597	0	4	2.8e5	7.1e3		c	g	-	-			
3	27.04 21:42	11.5	0.3	10.8	387	1472	68	99	1.1e6	1.3e4		c	c	c	c			
4	04.05 16:32	8.6	0.5	3.6	555	1329	0	4	5.4e5	5.9e3		c	c	-	-			
5	08.05 13:37	22.2	1.3	6.0	1820	7355	327	1018	2.0e6	4.4e4	A	c	c	c	c	t	t	2 (20)
6	20.05 00:42	31.1	1.4	6.0	2306	1111	3586	1406	2.7e6	2.9e4	A	c	c	c	c	-	t	3 (30)
7	25.05 17:02	4.6	0.5	6.0	139	1	0	2	4.4e5	3.6e4	N	c	g	g	g			
8	28.05 07:17	11.7	1.1	8.4	814	379	439	165	8.8e5	1.7e4		c	c	g	-			
9	29.05 17:57	4.7	1.3	25.2	865	960	136	606	4.3e5	1.9e4	N	c	c	g	c			
10	06.06 10:12	7.3	0.9	4.8	437	5	0	5	9.7e5	1.9e4	A	g	g	g	g	t	b	3 (30)
11	10.06 11:52	38.2	0.9	9.6	5378	7034	951	1721	8.7e6	2.8e4	N	c	c	c	c			
12	15.06 17:57	21.5	8.6	58.8	5230	3379	586	4109	3.5e6	8.9e4	A	c	c	g	c	t	t	3 (30)
13	01.07 18:07	11.6	1.8	27.6	988	249	0	30	7.7e5	6.5e4	A	c	c	c	g	t	b	3 (30)
14	06.07 09:42	8.9	1.8	10.8	642	98	0	9	9.7e5	8.7e4	A	c	g	g	g	-	b	3 (30)
15	14.07 21:47	15.1	2.0	8.4	1185	1547	33	111	7.0e5	2.3e4	N	c	c	g	g			
16	28.07 12:17	14.5	0.8	10.8	427	94	0	12	2.8e5	1.7e4		g	g	g	g			
17	06.08 07:07	16.7	1.1	24.0	865	531	0	117	4.9e5	1.6e4		c	c	g	g			
18	10.08 03:42	25.4	2.0	58.8	2087	2349	144	614	1.6e6	1.3e4		c	c	c	c			
19	18.08 22:32	18.8	2.0	50.4	1311	1099	28	224	8.6e5	1.2e4		g	g	g	g			

Table A7: Overview over the transformation product concentrations measured at the different sampling sites. To calculate mean concentrations, we replaced concentrations below the limit of quantification (LOQ) by zero (lower value reported) and by the LOQ (higher value reported). The transformation product pattern shown here is most likely caused by the low number of transformation products analysed and does not allow for a general conclusion on transport processes involved. I1-I4: inlets, CS: collector shaft, ST: stream.

Site	I1	I2	I3	I4	CS	ST
Mean transformation product concentration (ng L^{-1})	271-301	25-56	36-66	10-43	714-716	1003-1006
Maximal transformation product concentration (ng L^{-1})	7300	870	550	180	5200	5500
Transformation product with highest concentration	Meta-mitron-Desamino	CT-TP-R471811	CT-TP-R471811	Meta-mitron-Desamino	Meta-mitron-Desamino	CT-TP-R471811

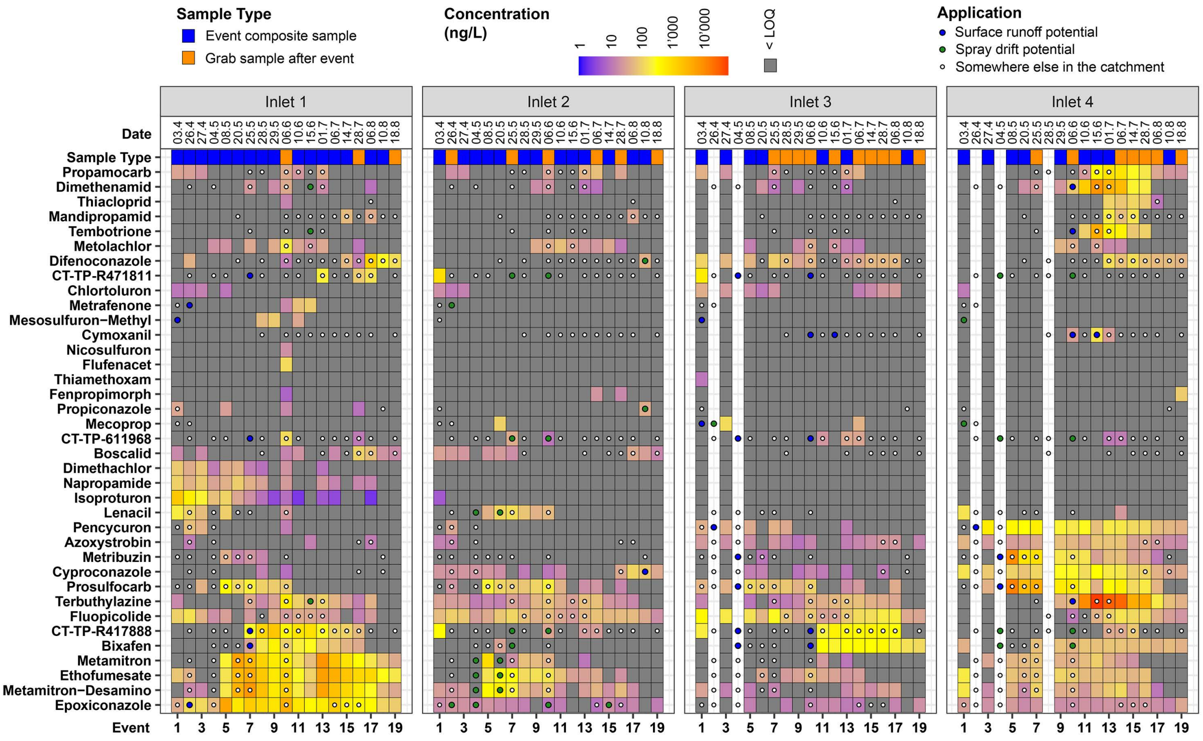

Figure A18: Concentrations c (ng/L) measured in inlets for event 1 (3 April 2019) to 19 (18 August 2019) for all substances that were found in inlets. White rows indicate that no sample was taken. In the first column, the sample type is indicated. In the remaining columns, substances are clustered by the concentrations measured. Coloured dots indicate that the particular substance was applied in the period between the respective and the previous event. Dot colours specify the potential transport processes. LOQ: Limit of quantification.

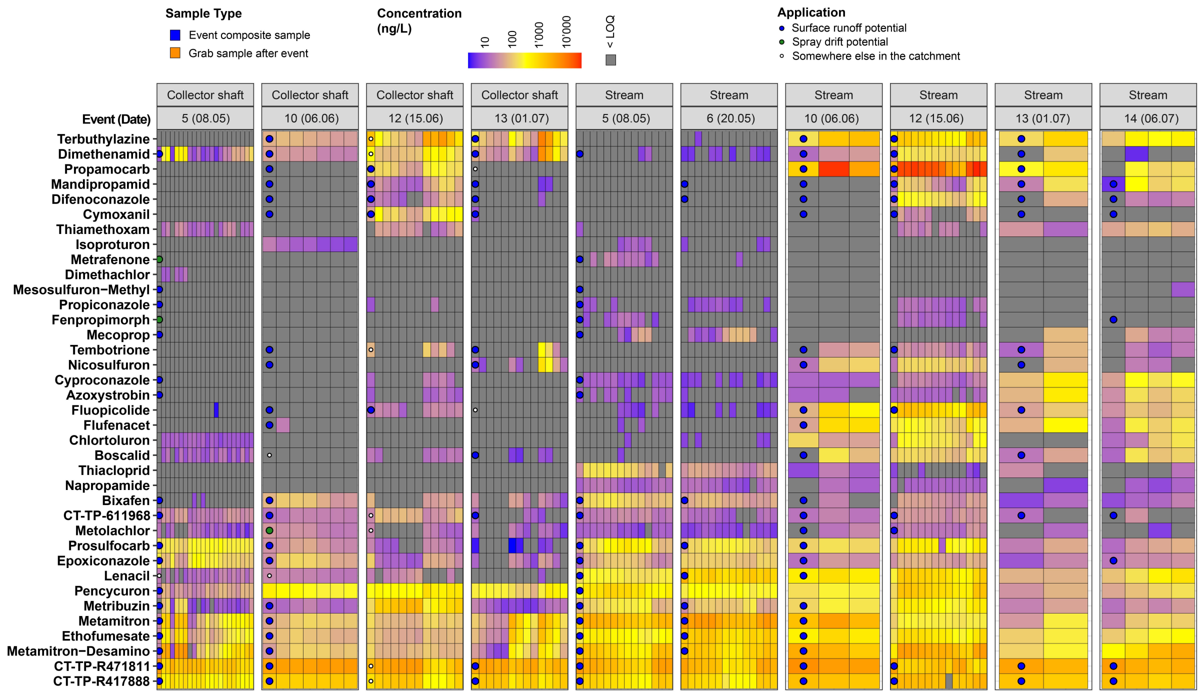

Figure A19: Concentrations c (ng/L) measured in the collector shaft (event 5, 10, 12, and 13) and in the stream (events 5, 6, 10, 12, 13, 14) for all substances found at one of these two sampling sites. Measurements of events 10, 13, and 14 in the stream originate from backup samples of the cantonal authorities. LOQ: Limit of quantification.

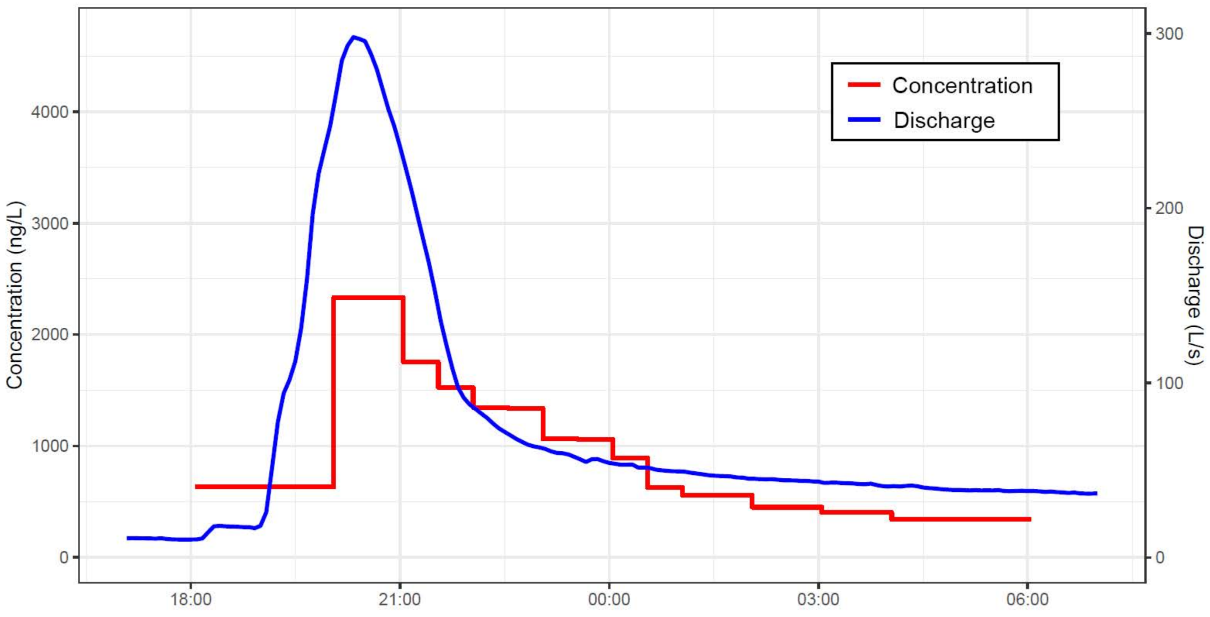

Figure A20: Discharge and terbuthylazine concentration in the stream during event 12 (07.08.2019 to 08.08.2019).

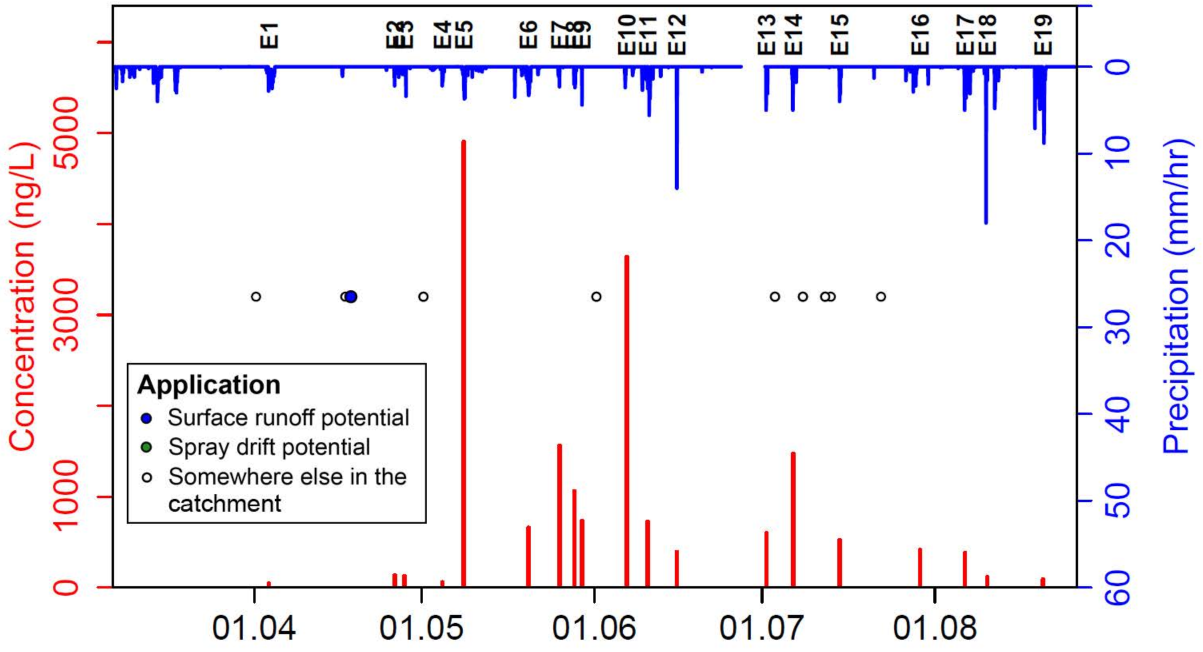

Figure A21: Concentrations of epoxiconazole in inlet 1, during the sampled events (E1 to E19).

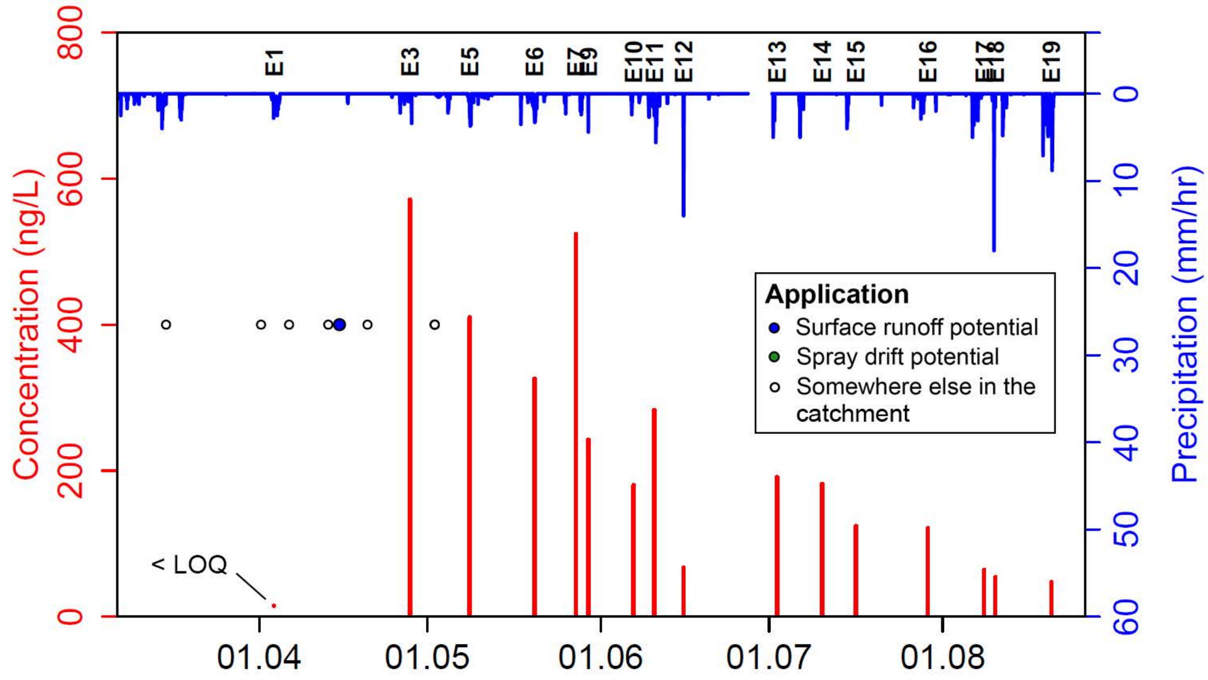

Figure A22: Concentrations of pencycuron in inlet 4, during the sampled events (E1 to E19). During E2, E4, and E8, no samples were taken. No pencycuron was found in the first sample (i.e. the concentration was smaller than the limit of quantification (LOQ) of 15 ng/L).

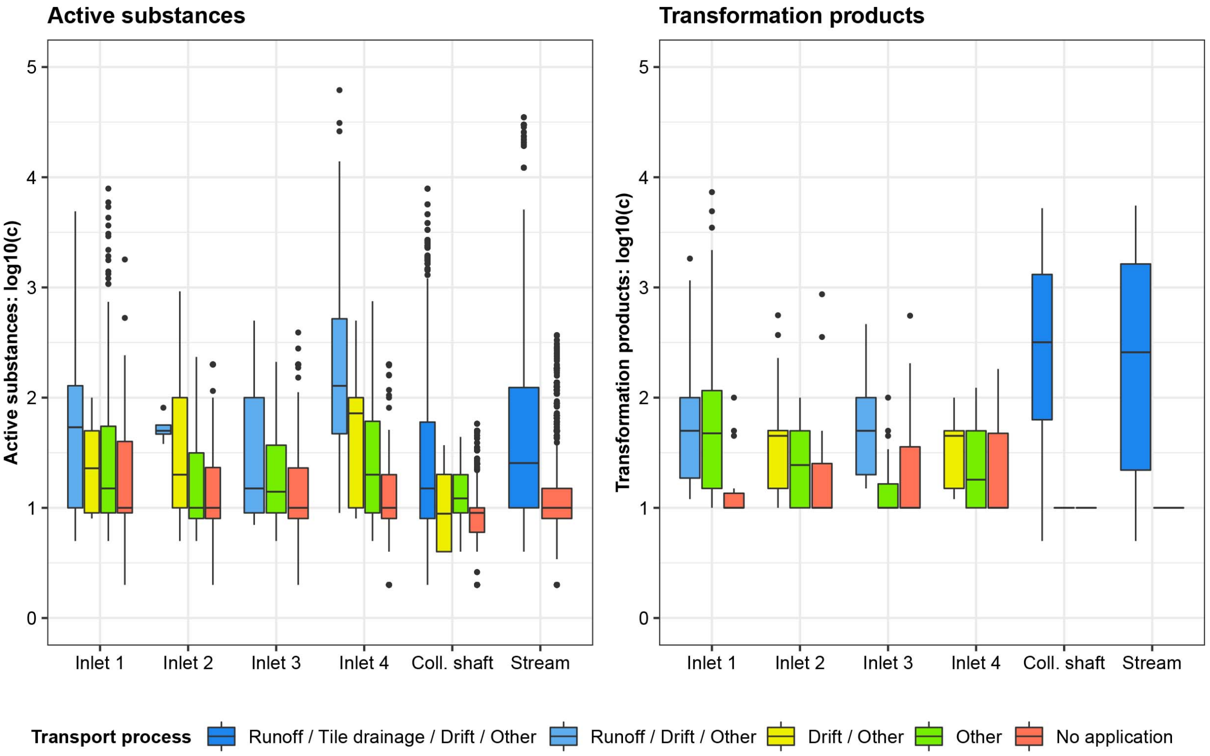

Figure A23: Distribution of pesticide and transformation product concentrations for all sampling sites. Concentrations are assigned to possible responsible transport processes. For substances below the limit of quantification (LOQ), the LOQ was used for the analysis.

Table A8: Result of the linear mixed model. Estimates, t values, and p values are given for each explanatory variable. An explanation of the column "estimates" is provided in the following. For the calculation of p-values, a Kenward-Roger approximation was used for calculating the degrees of freedom. The proportion of variance explained by the fixed factors alone (marginal R2) was 0.25. The proportion explained by the fixed and random factors (conditional R2) equalled 0.48.

**Explanatory variable**	**Abbreviation**	**Unit**	**Estimates:** Mean [confidence interval: 2.5% – 97.5%])	**t value**	**p value**
Intercept	Int.	-	+ (1.4 [1.0 – 1.9])	6.24	< 0.001
Time since application	t_{appl}	days	– (2.4 [3.9 – 1.0]) · 10^{-3}	-3.35	0.001
Amount of pesticide applied per area (log10)	$\log_{10}(m_{appl})$	g ha^{-1}	+ (2.2 [1.5 – 2.9]) · 10^{-1}	6.24	< 0.001
Freundlich adsorption coefficient normalized to organic carbon content (log10)	$\log_{10}(K_{foc})$	mg L^{-1}	– (2.2 [3.2 – 1.2]) · 10^{-1}	-4.39	< 0.001
Half-life in water	$DT_{50,\ water}$	days	+ (3.2 [1.1 – 5.3]) · 10^{-3}	2.98	0.003
Half-life in soil	$DT_{50,\ soil}$	days	+ (1.1 [0.6 – 1.6]) · 10^{-3}	4.56	< 0.001
Moderate estimate of the discharge in the inlet during the event (log10)	$\log_{10}(Q_{mod})$	L	+ (1.1 [-4.0 – 6.0]) · 10^{-2}	0.45	0.654
Potential transport processes involved	$p_{transport}$	-	– (5.1 [6.3 – 4.0]) · 10^{-1}	-8.54	< 0.001

To improve the understandability of Table A8, the meaning of the "estimates" column is explained in the following. This column represents the mean estimates of the fixed effects of the linear mixed model and their confidence interval (2.5% to 97.5%). These effects corresponds to an intercept for row 1, to a slope for rows 2 to 7, and to a categorical variable effect for row 8.

In the following, the meaning of these estimates is explained on the example of the variable "time since application" (row 2). In a mathematical notation, our mixed model can be written as follows:

$$\log_{10}(c) = \text{Intercept} + m_1 \cdot t_{appl} + m_2 \cdot \log_{10}(m_{appl}) + m_3 \cdot \log_{10}(K_{foc}) + \cdots \quad \text{(A5)}$$

Where m_1, m_2, m_3, … are the estimated slopes, c is the pesticide concentration (ng/L) and t_{appl} is the time since application (days). As shown in Table A8, the mean estimate of the slope m_1 equals $-2.4 \cdot 10^{-3}$ day^{-1}. This means, that the logarithm ($\log_{10}$) of the measured concentration (ng/L) is expected to decrease on average by a factor of $-2.4 \cdot 10^{-3}$ per day after application.

Table A9: Ratio between pesticide loads in inlets and the stream (moderate estimates, eq. A3.1 and A3.2). In square brackets, the minimum and high estimates are given. Columns show the ratios measured for the sampled inlets, and the ratios resulting from extrapolating the measurements to the entire catchment using three different methods, i.e. proportional to the road area, the agricultural area, and the number of inlets.

	Sampled inlets (I1-I4)	Extrapolation to entire catchment		
		Road area	Agri. area	Number of inlets
Mean of single substance load ratios $r_{f,\mu,subst}$	1.8% [0.77%; 3.7%]	61% [27%; 126%]	30% [13%; 64%]	70% [30%; 144%]
Ratio of load sums $r_{f,\mu,sum}$	0.29% [0.24%; 0.52%]	10% [8.5%; 18%]	5.1% [4.2%; 9.1%]	12% [9.7%; 21%]

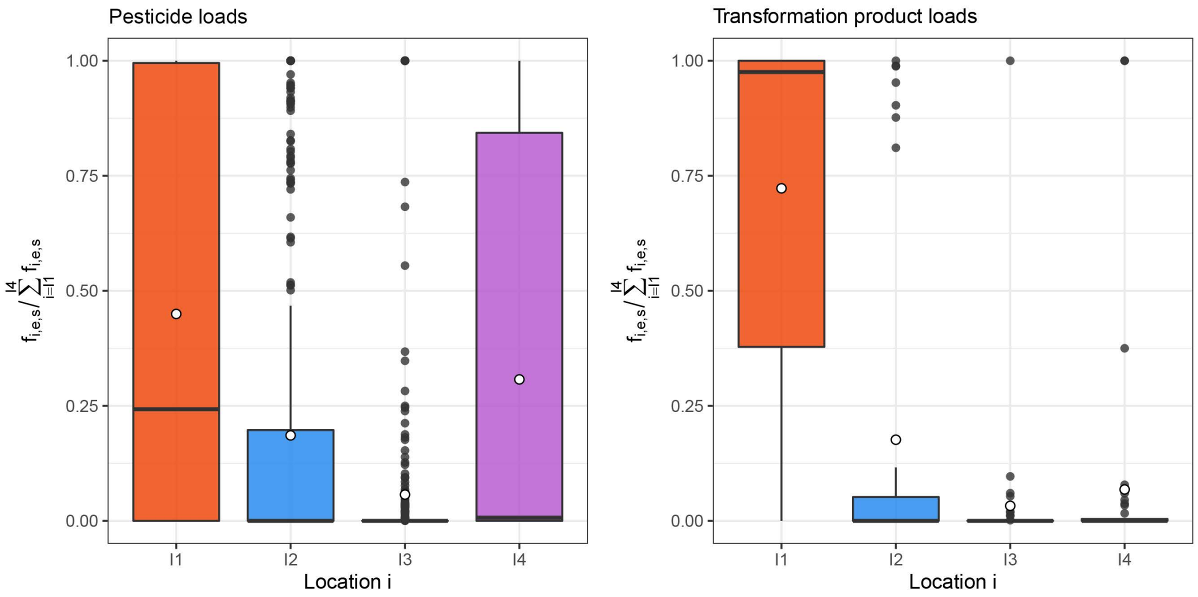

Figure A24: Relative loads per inlet compared to the loads transported through the four measured inlets. Left: Pesticides, right: transformation products. The transformation product pattern shown here is most likely caused by the low number of transformation products analysed and does not allow for general conclusions on the transport processes involved.

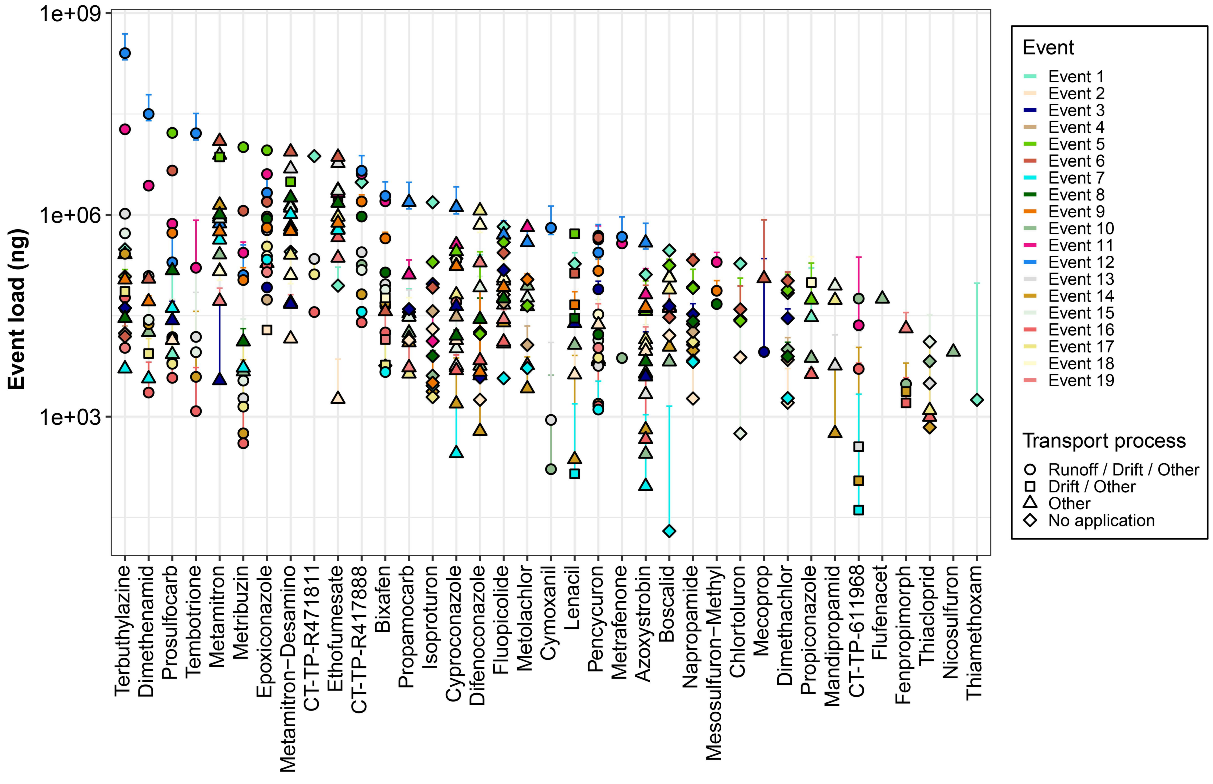

Figure A25: Sum of loads in the four inlets per substance and event. Only loads > 0 ng are shown.

References

Fuka, D. R., Walter, M. T., Archibald, J. A., Steenhuis, T. S., and Easton, Z. M.: EcoHydRology: A Community Modeling Foundation for Eco-Hydrology, R package version 0.4.12.1., https://CRAN.R-project.org/package=EcoHydRology, 2018.

Lyne, V., and Hollick, M.: Stochastic Time-Variable Rainfall-Runoff Modeling, Australian National Conference Publication, Perth, Australia, 1979.

Nathan, R. J., and Mcmahon, T. A.: Evaluation of Automated Techniques for Base-Flow and Recession Analyses, Water Resour Res, 26, 1465-1473, https://doi.org/10.1029/WR026i007p01465, 1990.

Schönenberger, U., Patrick, M., Wullschleger, S., and Stamm, C.: A water-level proportional water sampler for remote areas, Zenodo, 11, https://doi.org/10.5281/zenodo.4280534, 2020.

Swisstopo: swissTLM3D – The Topographic Landscape Model, Federal Office of Topography [data set], https://doi.org//10.5169/seals-236706, 2020.
